# In-class AI usage and university students’ attitudes: The mediating role of perceived learning engagement benefits

**DOI:** 10.1371/journal.pone.0355490

**Published:** 2026-08-04

**Authors:** Wenqiang Fan, Jiaqi Fang, Yanlin Sun, Xinrui Xie, Yaodong Li

**Affiliations:** 1 School of Humanities & Social Sciences, Beihang University, Beijing, China; 2 School of Beijing, Beihang University, Beijing, China; Shandong University, CHINA

## Abstract

With the widespread adoption of artificial intelligence (AI), the usage of various AI tools by university students to support learning has become increasingly common in classroom settings. Focusing on routine in-class AI usage and learning engagement, this study explores the mediating role of perceived learning engagement benefits in linking students’ AI usage behavior to their attitudes, and investigates whether its mediating effects differ across various dimensions of learning engagement. Based on data from 287 questionnaires, structural equation modeling and Bayesian analysis were used to test the proposed hypotheses. The results indicate that in-class AI usage was associated with students’ attitudes both directly and also indirectly by enhancing cognitive engagement and emotional engagement, both of which exhibited comparable mediating effects. In general, the university students exhibited positive attitudes toward in-class AI usage, and these attitudes were positively associated with their perceived learning engagement benefits. These results suggest that the value of AI in classroom settings may relate not only to learning efficiency, but also to students’ learning experiences, particularly in terms of perceived cognitive and emotional engagement benefits. Notably, although in-class AI usage was positively associated with all dimensions of perceived engagement benefits, perceived behavioral engagement benefits showed no significant indirect effect on attitudes. This null finding warrants further exploration in future research.

## 1. Introduction

In recent years, generative artificial intelligence (GenAI) has rapidly permeated all areas of higher education, to such an extent that it is now an indispensable part of university students’ academic lives [1,2]. GenAI tools such as ChatGPT and DeepSeek are now widely used by university students for tasks ranging from drafting papers and solving complex problems to the generation of creative content [3,4]. Although GenAI offers unprecedented opportunities for improving learning efficiency and accessibility, its impact on students’ learning is known to be far from unidimensional [1,5]. On the one hand, GenAI can serve as a powerful cognitive scaffold that empowers personalized learning and academic success [6,7]; on the other hand, it can undermine critical thinking, lower cognitive engagement, and foster passive learning behaviors [8,9]. GenAI thus has complex and multifaceted associations with learning, pointing to a need for a detailed and in-depth examination of its educational implications.

The classroom is the primary setting where structured learning takes place, and the teaching interactions that occur within this space is significantly associated with students’ academic development. In traditional university classrooms, insufficient student engagement has long been a concern [10–12]. In the absence of immediate feedback and personalized encouragement, students’ interest in learning and their self-confidence are likely to be adversely affected [13,14]. By contrast, GenAI technology offers core capabilities such as natural language interaction, real-time feedback, and personalized adaptation, and it thus provides a new pathway for tackling the challenges of traditional teaching. Classroom use of AI is becoming increasingly prevalent, and its impact is becoming more pronounced [15,16]. Whether introduced by instructors as a teaching aid or independently adopted by students, AI technology is currently reshaping the ecological structure of classroom learning [17]. A new triadic, interactive model encompassing students, teachers, and AI is gradually replacing the traditional dyadic, teacher-student interactive model as student-AI interaction becomes a primary form of classroom participation [18].

Against this backdrop, can the integration of AI into classroom learning promote students’ learning engagement? Studies on the relationship between AI usage and learning engagement have mostly been conducted in experimental or quasi-experimental settings with specific teaching interventions [19], or have not been confined to in-class learning contexts (e.g., [20]). With the ongoing popularization of AI usage, university students are increasingly turning to AI tools on their own initiative, even in the absence of conscious guidance from teachers, to assist their learning in classrooms [21,22]. The current study directly focuses on in-class AI usage and in-class learning engagement. It explores the status quo of university students’ attitudes toward in-class AI usage and their perceptions of whether in-class AI usage can enhance learning engagement, whether learning engagement mediates the relationship between AI usage behavior and attitudes, and whether there are differences in those mediating effects across the various dimensions of learning engagement. This study aims to provide empirical evidence and decision-making reference for the rational understanding and effective guidance of AI technology use to empower classroom teaching.

## 2. Literature review

With the rapid application of GenAI in the field of education, as represented by ChatGPT, the question of how AI relates students’ learning engagement has become a new focus of research. The mainstream scholarly perspective conceptualizes learning engagement as a multidimensional construct that encompasses behavioral engagement, emotional engagement, and cognitive engagement [23]. Focusing on the relationship between AI usage and learning engagement, this section summarizes the main findings of research to date, and the gaps in that research.

### 2.1. Main findings

The association between AI and learning engagement has been found to be predominantly positive. Relevant studies have generally recognized the potential of AI technologies for facilitating learning engagement [4,15,16,20,24–28]. From the cognitive perspective, AI fosters higher-order thinking, the use of metacognitive strategies, and self-regulation through personalized task delivery, immediate feedback, and problem-solving support [7,24,29–32]. By providing immediate feedback as well as support for concept clarification, idea generation, and problem decomposition, AI helps lower the initial cognitive load of learning tasks, thereby helping students to engage in a higher level of cognitive processing [33,34]. To verify the accuracy of AI outputs, students must take the initiative to compare them with course knowledge and consult authoritative literature, thereby stimulating their critical thinking [35]. In terms of behavioral engagement, AI significantly improves students’ task participation, frequency of resource utilization, and persistence in learning behaviors [7,31,36,37]. In terms of emotional engagement, AI fosters positive emotional states by enhancing learning motivation, interest, self-confidence, and satisfaction [6,7,22,27,37–39]. GenAI has characteristics such as immediate responses, low evaluation risk, and high controllability, which are believed to alleviate learning anxiety and enhance academic self-efficacy and which have particularly pronounced effects on students with weaker academic foundations or lower willingness to participate in class [20].

However, AI usage may also show a negative association with learning engagement, such as weakening cognition, reducing interactions, and triggering anxiety. When students excessively outsource the processes of thinking, reasoning, and decision-making to AI, their own level of cognitive efforts declines, thereby undermining their cognitive engagement and deep processing [36]. Over-reliance on AI is known to give rise to AI burnout and the diminishment of critical thinking [8]. The introduction of GenAI into the classroom may also trigger the problem of attention competition. When students engage in interactions with AI at the same time as teacher-led instructional activities in class, their limited cognitive resources become fragmented, resulting in decreased peer interactions and engagement with instructional content [29]. Reliance on AI diminishes proactive learning behaviors such as note-taking and independent thinking [40]. AI usage may lead to technology-related anxiety, a sense of dependence, or emotional isolation [3,8]. Some students experience frustration and a crisis of trust when confronted with inaccuracies and technical limitations in AI-generated content [39]. In addition, the “novelty effect” of AI tools diminishes over time, with learning engagement generally showing a declining trend towards the end of the semester [9].

These different relationships between AI usage and learning engagement — some positive, and some negative — may be related to contextual and cognitive factors. Teacher guidance and structured instructional design are known to be key to ensuring that AI actually boosts learning engagement [15,41]. When clear learning objectives, usage guidelines, and teacher guidance are provided, AI-assisted learning can significantly enhance students’ task persistence, in-class participation, and engagement in problem-solving [17,42]. Guo et al. [4] showed that the positive effects of AI on cognitive engagement and emotional engagement are only significant in the context of “high academic challenge + high teacher support”; whereas in the context of “low challenge + high teacher support”, AI usage reduces behavioral engagement and motivation. Wang et al. [29] found that classroom climate, AI literacy, and psychological resilience all have significant positive predictive effects on students’ in-class engagement, underscoring the crucial role of environmental and cognitive factors in promoting students’ active participation in the learning process. Ji and Fang [43] argue that the associations of AI usage with student engagement is moderated by learning goals: while students aiming to improve their competence enhance cognitive engagement by using AI to expand knowledge boundaries and validate viewpoints, those who simply aim to avoid falling behind tend to undermine their behavioral engagement (e.g., in-class interaction and post-class review) by relying on AI for ready-made answers.

### 2.2. Research gaps

Existing studies, especially experimental and quasi-experimental ones, have mostly investigated AI use under teacher intervention [4,20,25], but have paid insufficient attention to scenarios in which students regularly use AI in class on their own initiative. With the popularization of AI technologies, the independent use of various AI tools by students to support learning has become commonplace in university classrooms, even in the absence of deliberate teacher guidance. More exploration is needed regarding the relationship between AI usage and learning engagement in contexts without specific teacher intervention.

Moreover, some studies have mostly investigated learning engagement comprehensively across in-class and out-of-class contexts, rather than strictly confining the research scope to in-class settings (e.g., [20]). This limits these studies’ contribution to our understanding of the enhancement of in-class learning engagement. It is, therefore, very much necessary to distinguish between in-class and out-of-class AI usage behaviors, so as to gain a clearer understanding of the associations of AI intervention with students’ cognitive, behavioral, and emotional engagement in the classroom.

In addition, there is a lack of research examining learning engagement in conjunction with students’ attitudes toward in-class AI usage. Most relevant studies have treated learning engagement as an outcome variable, and only a few have examined it as a mediator in the relationship between AI usage and other variables [17,42,44]. Students’ attitudes toward in-class AI use serve as a key link connecting AI technology, teaching practices, and learner experiences. However, there is a lack of empirical research addressing the question of whether enhanced learning engagement can facilitate the formation of positive attitudes.

Based on the above research gaps, the current study investigates the association between routine in-class AI usage and learning engagement and the mediating role of enhanced learning engagement in the link between AI usage behavior and attitude.

## 3. Hypothesis and research model

### 3.1. Students’ In-Class AI usage and attitude

Attitude commonly influences behavior, and, conversely, individuals’ actual behaviors can shape or reinforce their cognitive and emotional tendencies toward such behaviors [45]. According to the Self-Perception Theory [46], when students use AI to assist their learning in class, they perceive, adjust, or consolidate their own attitudes. Sustained usage behaviors may trigger a need for cognitive consonance, prompting them to align their attitudes with their behaviors in order to preserve consistency [47]. In educational contexts, effective user experiences can help students understand the functionality and value of AI, which can significantly enhance their acceptance and positive evaluations of it [48,49]. Especially for students whose attitudes are initially ambiguous or neutral, behavioral experience can be a key factor in shaping their attitudes. Frequent usage can also reduce unfamiliarity and anxiety toward AI technology and foster a sense of control, thus facilitating the development of positive attitudes [20,50]. Therefore, the following hypothesis is proposed:

**Hypothesis 1 (H1):** Students’ in-class AI usage is positively associated with their attitudes toward in-class AI usage.

### 3.2. In-Class AI usage and learning engagement

By providing personalized learning support, adaptive learning pathways, and immediate explanations, AI can stimulate students to engage in cognitive activities, thereby deepening their understanding, enhancing their problem-solving abilities, and fostering higher-order thinking [4,6,7,31,36,44]. In class, when students encounter difficulties such as conceptual confusion, misunderstandings regarding formulas, or gaps in their connections among different pieces of knowledge, they can use AI’s immediate response function to quickly obtain precise explanations and remove learning obstacles in a timely manner, without having to wait to consult the teacher after class or look through voluminous reference materials. This immediacy not only prevents students’ understanding of subsequent content from being hindered by accumulated doubts, but also enables students to maintain the continuity of their thinking and their desire to explore at all times [51]. When students can address their questions promptly and explore diverse dimensions of knowledge, their willingness to engage in active inquiry increases significantly [52], thereby achieving an effective improvement in their in-class cognitive engagement. Hence, the following hypothesis is proposed:

**Hypothesis 2 (H2):** Students’ in-class AI usage is positively associated with their perceived cognitive engagement benefits.

Behavioral engagement is manifested as classroom participation, task completion, and interaction frequency [23,53]. AI tools can stimulate interactive behaviors that enhance students’ learning enthusiasm and classroom participation [2,29,36,37,53,54]. In class, when students can turn to AI for help, they are more inclined to answer the teachers’ questions, participate in group discussions, and complete in-class tasks. Hence, the following hypothesis is proposed:

**Hypothesis 3 (H3):** Students’ in-class AI usage is positively associated with their perceived behavioral engagement benefits.

Emotional engagement involves having interest, motivation, and a sense of pleasure in learning, as well as a sense of belonging [6,23]. AI can improve students’ emotional experiences and enhance their emotional engagement through personalized feedback, and interactivity [6,7,9,20,39,55]. In class, AI neither belittles students for asking simple questions nor judges them for holding divergent viewpoints; instead, it consistently listens to their thoughts and provides patient feedback and suggestions. By creating an inclusive interactive atmosphere, AI can eliminate students’ sense of fear and inferiority, thereby creating a safe learning environment [38]. Non-judgmental feedback can reduce students’ learning anxiety, enhance their sense of autonomy, and foster a sense of accomplishment [56], thereby improving their emotional engagement. Hence, the following hypothesis is proposed:

**Hypothesis 4 (H4):** Students’ in-class AI usage is positively associated with their perceived emotional engagement benefits.

### 3.3. The mediating role of perceived learning engagement benefits

When students perceive that using AI for learning effectively helps them understand complex concepts, solve difficult problems, and deepen their thinking (i.e., cognitive engagement), they will evaluate the usefulness of AI from a perspective of instrumental rationality [4]. According to the Technology Acceptance Model [57,58], users’ attitudes toward technology are primarily influenced by perceived usefulness, that is to say, the degree to which the technology can help them achieve their goals. When AI has proven itself to be an effective tool for achieving cognitive goals, students’ attitudes toward it become more positive and accepting [31,36]. In class, when students engage more actively in deep cognitive activities using AI tools, they can directly perceive the value of AI in supporting their reflective and critical learning, improving their cognitive efficiency, and helping them break through bottlenecks in their thinking. This in turn strengthens their perception of AI as a useful tool and ultimately fosters the formation of positive attitudes. Hence, the following hypothesis is proposed:

**Hypothesis 5 (H5):** Perceived cognitive engagement benefits mediate the relationship between students’ in-class AI usage and their attitude toward it.

When students exhibit a high level of behavioral engagement, they use AI more proactively for participating in classroom tasks. If they find that AI can help them complete these tasks more smoothly, they will gain a more intuitive sense of its role in quickly resolving problems, which will significantly elevate their perceived usefulness of AI use, leading to positive evaluations of AI. Individuals tend to seek rational justifications for their sustained behaviors and thus form attitudes consistent with those behaviors [47]. High-frequency usage lowers barriers to use and enhances familiarity and a sense of control, leading to positive evaluations [59]. In class, the more frequently students use AI to interact with teachers, collaborate with peers, and participate in classroom activities, the more inclined to identify with such behavior. Sustained interaction with their teachers and peers itself contributes to a positive learning experience, strengthening students’ reliance on and affinity for AI tools [36,37,53] and serving as the foundation for the formation of attitudes. Based on this, the following hypothesis is proposed:

**Hypothesis 6 (H6):** Perceived behavioral engagement benefits mediate the relationship between students’ in-class AI usage and their attitudes toward it.

When students experience enjoyment, a sense of accomplishment, competence, and support (rather than frustration) while using AI, they will develop positive emotional connections with it [39,55]. According to the Affective Events Theory [60], such positive emotional experiences directly influence individuals’ overall evaluations of the objects associated with those experiences. In class, AI tools enhance students’ recognition of the value of AI usage by boosting their motivation and self-confidence [7,27]. Pleasant, engaging, and supportive emotional experiences prompt students to link AI to positive emotional labels [6], thereby forming a more positive and accepting attitude toward AI usage. Based on this, the following hypothesis is proposed:

**Hypothesis 7 (H7):** Perceived emotional engagement benefits mediate the relationship between students’ in-class AI usage and their attitudes toward it.

### 3.4. Research model

**Fig 1:**
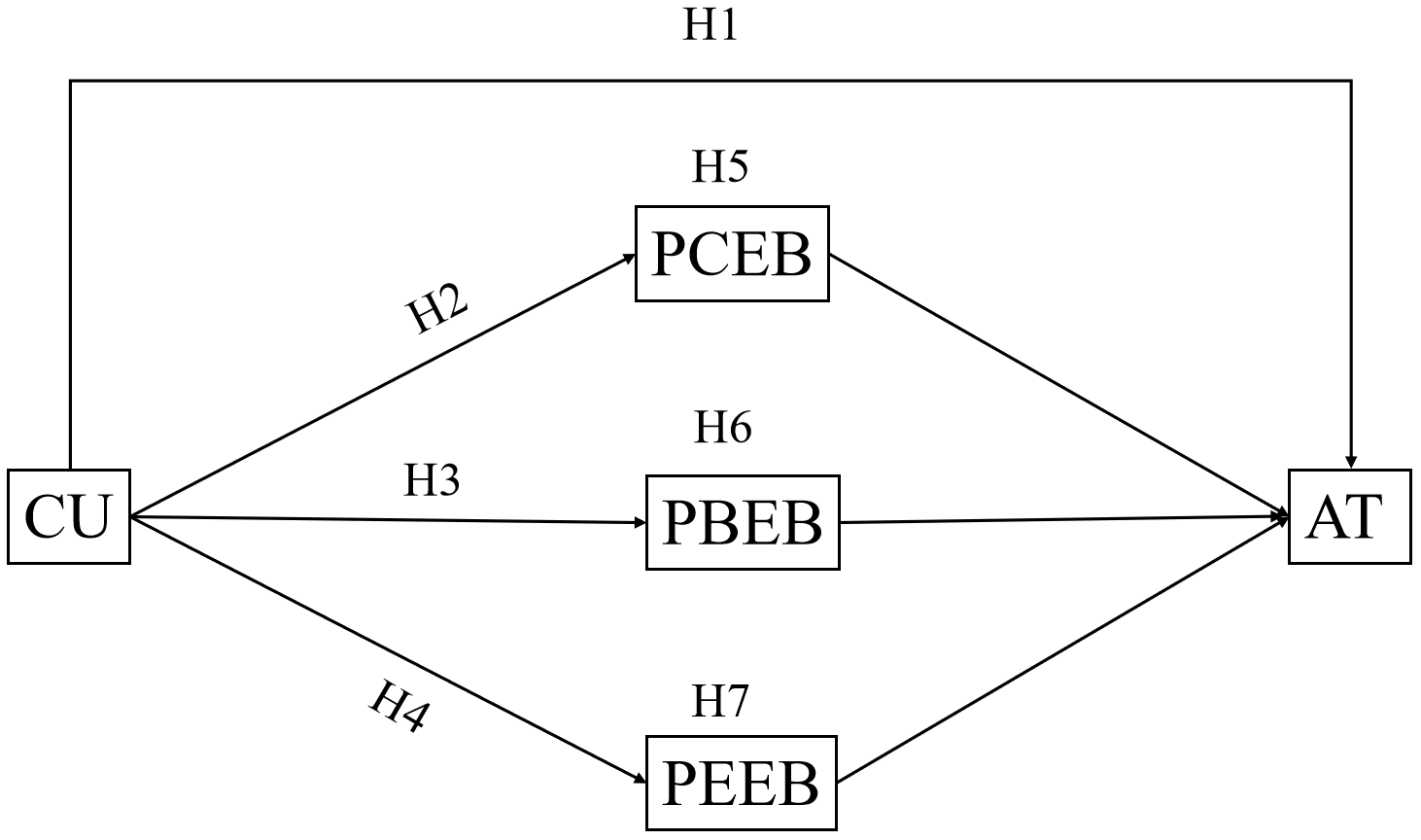
The research model. Note: CU: In-class AI usage; PCEB: Perceived cognitive engagement benefits; PBEB: Perceived behavioral engagement benefits; PEEB: Perceived emotional engagement benefits; AT: Attitude toward in-class AI usage.

The hypotheses enumerated above collectively constitute the complete research model of the mediating pathway of “Usage-Engagement-Attitude”, as illustrated in Fig 1.

Drawing on Self-Perception Theory [46], Cognitive Consistency Theory [47], and Affective Events Theory [60], a three-dimensional theoretical framework is established to elaborate the multifaceted mechanisms linking students’ in-class AI use to the formation of their AI attitudes. Instead of offering competing interpretations, the three theories correspond to distinct psychological processing depths, representing complementary interpretive angles: surface-level behavioral observation (Layer 1), middle-level cognitive reconciliation (Layer 2), and deep-level affective response (Layer 3).

Layer 1 posits that students initially form AI attitudes by observing their own in-class AI use behaviors, including relying on AI for problem-solving, drafting, and information retrieval. Without strong preexisting attitudes, students function as “naïve self-observers”: they infer a positive stance from frequent, voluntary AI engagement, while limited or avoidant AI use signals negative dispositions. This layer depicts the foundational stage of attitude formation, in which behavioral observations shape attitudes when internal psychological cues remain weak or ambiguous.

Layer 2 emerges when students encounter psychological tension between their preexisting beliefs and observations of AI usage. To restore cognitive consistency, individuals engage in active reasoning, rationalization and reinterpretation—for instance, perceiving AI as a valid learning tool and justifying their AI use as consistent with academic objectives. This layer evolves basic behavioral inference into a stable, cognitively refined stance, wherein attitudes move beyond superficial observation to conscious cognitive structuring for dissonance reduction.

Layer 3 addresses how emotionally charged encounters with AI in classroom contexts shape affective attitudes. Specific events—such as an AI delivering a sudden breakthrough, making an embarrassing error, or receiving instructor praise—trigger immediate positive or negative emotional reactions. Over time, these episodic affective experiences consolidate into trait-like attitudes toward AI, which further shape subsequent behavioral interpretations and cognitive rationalizations rooted in the preceding layers. This framework thereby provides an integrated conceptual approach to understanding AI-related attitudes, ranging from behavioral inference and cognitive integration to emotional internalization.

Based on the above theoretical framework, this study proposes differential mediation effects across diverse engagement dimensions. First, high cognitive engagement is less constrained by external conditions and more strongly associated with intrinsic motivation, thereby fostering positive attitudes toward AI use. This study predicts that elevated cognitive engagement encourages learners to actively compare AI utilization with their personal cognitive logic, adjust their own attitudes, and attain a higher degree of cognitive alignment by mitigating cognitive dissonance. Furthermore, cognitive engagement assists individuals in regulating affective experiences during human–AI interactions: high cognitive engagement amplifies positive emotions and attenuates negative emotional responses. Overall, perceived cognitive engagement benefits are closely linked to self-attribution, cognitive consistency, and emotional transformation, and are predicted in this study to serve as a core and robust mediating variable in shaping learners’ attitudes toward AI.

In terms of perceived emotional engagement benefits, AI usage is closely associated with various affective events: effective AI support is expected to correlate with pleasure, whereas AI malfunction is hypothesized to link to frustration. As a key mediator, perceived emotional engagement benefits are anticipated to correlate with users’ perceptions of these affective events and experience-congruent attitudes. They are further predicted to display associations with user attitudes, as well as accelerated attitude formation and higher attitude-behavior consistency. Overall, perceived emotional engagement benefits are predicted to be centrally associated with users’ attitudinal shifts and exert robust mediating effects. However, given the volatile nature of emotion, these perceived emotional benefits may be less stable than cognitive ones.

In terms of perceived behavioral engagement benefits, they are hypothesized to function as the most distal cue for individual self-perception. Behavioral inference tends to emerge primarily when internal psychological cues are weak or ambiguous. In classroom environments where affective and cognitive cues are highly accessible, perceived behavioral engagement benefits are predicted to exert weaker links to AI-related attitudes. As observable overt conduct, perceived behavioral engagement benefits bear limited connections with the core mediating constructs rooted in Cognitive Consistency Theory—such as perceived cognitive inconsistency and cognitive restructuring—and correlate weakly with attitudinal change. Meanwhile, perceived behavioral engagement benefits are expected to relate only to the external manifestations of affective events rather than individuals’ internal affective responses, with limited relevance to interindividual attitudinal differences.

It is important to note that, while this framework proposes multiple layers of psychological processing, the cross-sectional survey design does not allow for empirical tests of temporal sequence or causal direction among the dimensions. Accordingly, this theoretical framework should therefore be interpreted as a conceptual heuristic to map concurrent psychological channels rather than a temporally verified path structure.

## 4. Methods

### 4.1. Survey samples

Questionnaire data were collected from 287 students from H University, a research-intensive university in China renowned for its strength in the engineering disciplines. The participants were recruited using convenience sampling. An online questionnaire was disseminated via WJX.cn, a widely used online questionnaire platform in China. The questionnaire was advertised to undergraduate and postgraduate students in various schools and departments of H University via the social media application WeChat. Upon clicking the survey link, participants were presented with a description of the research for them to review, an assurance that their participation would be anonymous, and the specific survey questions. All respondents took part in this survey voluntarily. Of the participants, 169 were males and 118 were females; 180 were undergraduates and 107 were postgraduates; 202 had science and engineering backgrounds and 85 had humanities and social sciences backgrounds.

This study was conducted in accordance with the ethical principles of the Declaration of Helsinki. Formal ethical approval was waived by the School of Humanities and Social Sciences, Beihang University, as the research involved no greater than minimal risk and relied on an anonymous, voluntary survey that did not collect any sensitive or personally identifiable information. Prior to participation, informed written consent was obtained from all participants via an introductory statement in the online questionnaire, which explicitly detailed the study’s purpose, data usage, anonymity guarantees, and the participants’ right to withdraw at any time without consequences.

The data for this study were derived from a larger survey project reported in Fan et al. [61], which uses the same dataset but investigates distinct research questions concerning the mediating roles of cognitive relief and cognitive offloading. As such, the descriptive statistics and reliability estimates for the shared variables (in-class AI use and user attitudes) are identical to those reported in Fan et al. [61]; they are reproduced in Table 1– 5 for the reader’s convenience, with appropriate citation.

### 4.2. Instruments

The questionnaire used in this study collected participants’ demographic information, specifically their gender, grade (undergraduate or postgraduate), and major field (science and engineering or humanities and social sciences). It also included the following pre-established scales. The In-Class AI Usage Behavior Scale comprises five items, all scored on a five-point Likert scale (1 = Never, 2 = Occasionally, 3 = Moderately, 4 = Frequently, 5 = Very frequently). The Perceived Enhancement of Learning Engagement through In-Class AI Use Scale comprises 11 items designed with reference to the relevant literature [4,27,29,39,41,44,54,62]. It is divided into three sub-dimensions: perceived cognitive engagement benefits (four items), perceived behavioral engagement benefits (three items), and perceived emotional engagement benefits (four items). The Students’ Attitudes toward In-Class AI Use Scale consists of three items. Exploratory factor analysis (EFA) extracted a single factor with an eigenvalue of 2.443, accounting for 81.431% of the total variance. The standardized factor loadings of the three items ranged from 0.890 to 0.920, with no evidence of double loading (i.e., cross-loadings). Together, these indices indicate that the Students’ Attitudes toward In-Class AI Use Scale is suitable for use in subsequent research. The items are scored on a five-point Likert scale from 1 = Strongly disagree to 5 = Strongly agree. The items of each part were finalized after adjustments based on feedback from the researchers to ensure their applicability to the in-class AI tool usage. The English translations corresponding to the Chinese versions of the Perceived Enhancement of Learning Engagement through In-Class AI Use Scale items are provided in S2 File.

In-class AI usage (CU): This variable was used to measure the frequency of students’ behaviors of using AI to assist their learning in classroom settings. A higher score indicates more frequent AI use for various in-class learning activities, such as answering teachers’ questions, completing quizzes, finishing design or analysis tasks, and supporting group discussions and writing [61].

Perceived cognitive engagement benefits (PCEB): This variable was used to assess students’ perception of whether in-class AI usage enhances their cognitive engagement. A higher score indicates stronger student agreement that in-class AI usage helps boost their cognitive engagement, including their understanding of complex concepts, thinking deeply about course content, expanding their knowledge, and diversifying their ways of thinking.

Perceived behavioral engagement benefits (PBEB): This variable was used to assess students’ perception of whether in-class AI usage enhances their behavioral engagement. A higher score indicates that a stronger belief that in-class AI usage boosts their behavioral engagement, as manifested in ways such as interacting with teachers, engaging with peers, and participating in classroom activities.

Perceived emotional engagement benefits (PEEB): This variable was used to assess students’ perception of whether in-class AI usage improves their emotional engagement. A higher score indicates a stronger belief that in-class AI usage enhances their emotional engagement, as manifested in learning interest, learning self-efficacy, confidence in tackling difficult tasks, a sense of learning accomplishment, etc.

Attitudes toward in-class AI usage (AT): This variable was used to assess students’ attitudes toward in-class AI usage. A higher score indicates a more positive attitude, including approval of rational use of AI and recognition of its positive role in improving learning quality and efficiency [61].

Control variables: Demographic variables, such as gender, grade, and major field, were used as control variables.

### 4.3. Data analysis

The data were first cleaned to remove invalid responses. All 287 valid questionnaires contained complete responses, with no missing data on any scale item. Data analysis was then conducted using SPSS 25.0 and Mplus 8.3 statistical software. The specific procedures used were assessment of the reliability and validity of the scales, calculation of descriptive statistics of the questionnaire items, and testing of the research hypotheses using Structural Equation Modeling (SEM). The Bayesian analysis method was used in this study to test the mediating effects, as this method is more suitable for the analysis of relatively small sample sizes. Bayesian estimation was conducted using Mplus with its default diffuse priors. Specifically, factor loadings, path coefficients, and intercepts were assigned normal priors with a mean of 0 and a variance of 10¹⁰, while residual variances and covariances were assigned non-informative inverse-gamma priors. To assess the sensitivity of posterior inference to prior specifications, we re-estimated the model by replacing the priors for all structural path coefficients with weakly informative normal priors N (0, 1), keeping all other specifications unchanged. The results indicated that the posterior medians of the core paths changed by no more than 0.03, the 95% credible intervals showed substantial overlap, and the conclusions regarding statistical significance remained identical. Thus, the Bayesian inferences in this study are robust and not sensitive to the choice of priors.

In this study, Harman’s single-factor test was used to examine common method bias. An unrotated principal component analysis on all measurement items revealed five factors with eigenvalues greater than 1.0, with the first factor accounting for 38.114% of the total variance, which is below the recommended threshold of 50% [63]. This indicates that common method bias is not a serious concern in this study and is unlikely to substantially distort the relationships among the variables.

## 5. Results

### 5.1. Scale reliability

Cronbach’s α coefficients were used in this study to assess scale reliability (see Table 1). The coefficient values for all five constructs exceeded 0.8, indicating a high level of scale reliability.

**Table 1:** Cronbach’s α coefficients of the scales.

Variable	CU	PCEB	PBEB	PEEB	AT
Number of Items	5	4	3	4	3
Cronbach’s α	.843 a	.891	.821	.845	.884 a

Note: Symbol a refers to data obtained from Fan et al. [61].

### 5.2. Scale validity

The questionnaire used in this study was developed based on existing literature and then revised according to feedback from three researchers to ensure its content validity. Confirmatory Factor Analysis (CFA) was performed using Mplus 8.3 software. The results (see Table 2) indicate an overall good model fit: χ²/df = 2.618, CFI = 0.925 and TLI = 0.910, RMSEA = 0.075, and SRMR = 0.059. All these values meet the criteria for good model fit (χ²/df < 3, CFI > 0.90, TLI > 0.90, RMSEA < 0.08, and SRMR < 0.08) [64,65].

**Table 2:** Model fit indices.

Fit Indices	χ²/df	CFI	TLI	RMSEA	SRMR
Model Results	2.618	0.925	0.910	0.075	0.059

The internal quality of the measurement model was then evaluated. As shown in Table 3, the standardized factor loadings of all observed variables on their corresponding latent factors range from 0.641 to 0.875, and all are significant at the level of p < 0.001. The composite reliability (CR) values for each factor range from 0.832 to 0.896, and thus all of them exceed the threshold value of 0.70. The average variance extracted (AVE) values range from 0.521 to 0.723, and thus they all exceed the minimum standard of 0.50 [66,67]. These results indicate that the measurement model exhibits good convergent validity and that each item effectively reflects its corresponding latent constructs with high internal consistency [64].

**Table 3:** Results for the measurement model.

Construct	Items	Standardized-Factor loading	AVE	CR
CU	CU1	0.721	0.521 a	0.845 a
	CU2	0.730		
	CU3	0.731		
	CU4	0.729		
	CU5	0.697		
PCEB	CE1	0.828	0.682	0.896
	CE2	0.827		
	CE3	0.829		
	CE4	0.819		
PBEB	BE1	0.803	0.624	0.832
	BE2	0.873		
	BE3	0.683		
PEEB	EE1	0.641	0.590	0.851
	EE2	0.805		
	EE3	0.828		
	EE4	0.785		
AT	AT1	0.826	0.723 a	0.887 a
	AT2	0.849		
	AT3	0.875		

Note: Symbol a refers to data obtained from Fan et al. [61].

Discriminant validity analysis was also performed. All relevant values indicate acceptable discriminant validity for the scales (see Table 4).

**Table 4:** Discriminant validity analysis.

Construct	CU	PCEB	PBEB	PEEB	AT
CU	**0.722** a				
PCEB	0.342	**0.826**			
PBEB	0.275	0.390	**0.790**		
PEEB	0.379	0.610	0.551	**0.768**	
AT	0.443	0.635	0.323	0.619	**0.850** a

Note: Diagonal values in bold are the square roots of the AVE for each factor; Symbol a refers to data obtained from Fan et al. [61].

### 5.3. Descriptive statistics and correlation analysis

Table 5 presents the correlation matrix, means, and standard deviations for each construct. The mean values of the five constructs range between 3.27 and 4.16.

**Table 5:** Means, standard deviations, and correlations among the study variables.

Construct	CU	PCEB	PBEB	PEEB	AT
CU	1				
PCEB	.289**	1			
PBEB	.241**	.349**	1		
PEEB	.319**	.531**	.539**	1	
AT	.387** a	.566**	.295**	.531**	1
Mean	3.56 a	4.13	3.27	3.79	4.16 a
S.D.	0.828 a	0.684	0.831	0.718	0.630 a

Note: **p < 0.01; Symbol a refers to data obtained from Fan et al. [61].

Given the overrepresentation of science and engineering students in the sample, differential analyses were conducted on five core variables across the two academic disciplines. Only PCEB (higher among science and engineering students, p = .005, d = 0.36) and PBEB (higher among humanities and social sciences students, p = .034, d = 0.28) showed significant differences, but both had small effect sizes. No disciplinary differences were found for the remaining variables.

### 5.4. Hypothesis testing

In this study, the hypotheses were tested using SEM conducted in Mplus 8.3. Bayesian analysis was employed with the Markov Chain Monte Carlo (MCMC) method for parameter estimation. The model employed two parallel chains over 40,000 iterations, with the first 10,000 iterations used as burn-in. Posterior samples were saved at intervals of every 10 iterations. Convergence diagnostics indicated that the final value of the potential scale reduction factor (PSRF) was 1.009, and the PSR values for all parameters were close to 1.000. This is well below the strict threshold of 1.05, thus verifying the reliability of the subsequent parameter estimation results [68,69]. In addition, examination of the trace plots and autocorrelation plots for the parameters’ posterior distributions revealed no abnormal trends or high autocorrelation, further verifying the good model convergence. All analyses controlled for gender, grade, and major as covariates affecting the independent, mediating, and dependent variables.

**Fig 2:**
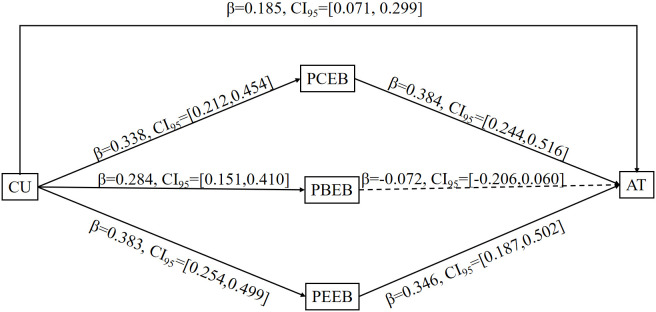
Mediating path diagram. Note: Solid lines indicate credible effects; dashed lines indicate non-credible effects.

The MCMC chains converged well (PSR ≤ 1.001), the 95% credible intervals had reasonable widths, with DIC = 11273.730 and pD = 81.603. These indices indicate that the current sample size (N = 287) was sufficient to support stable estimation of the model with 5 latent variables and 82 free parameters. The path coefficients are presented in Fig 2. The SEM results are summarized in Table 6. The results of the multiple mediating effect test are presented in Table 7.

**Table 6:** Standardized path coefficients of the SEM.

Paths	Coefficient estimates	S.E.	Lower 95%CI	Upper 95%CI
CU→AT	0.185	0.058	0.071	0.299
CU→PCEB	0.338	0.062	0.212	0.454
CU→PBEB	0.284	0.066	0.151	0.410
CU→PEEB	0.383	0.062	0.254	0.499
PCEB→AT	0.384	0.069	0.244	0.516
PBEB→AT	−0.072	0.068	−0.206	0.060
PEEB→AT	0.346	0.080	0.187	0.502

**Table 7:** Results of multiple mediating effect test (standardized).

Standardized effects	Effect size proportion	SE	Estimates	95% CI	
				Lower Limit	Higher Limit
Standardized total effects					
CU→AT	100%	0.057	0.335	0.231	0.454
Standardized direct effects					
CU→AT	55.2%	0.058	0.185	0.071	0.299
Standardized indirect effects					
CU→PCEB→AT	53.8%	0.029	0.100	0.053	0.164
CU→PBEB→AT	–	0.017	−0.015	−0.052	0.013
CU→PEEB→AT	54.8%	0.032	0.102	0.050	0.173
Total indirect effects	56.1%	0.039	0.188	0.119	0.273

As shown in Table 6, CU significantly and directly predicted AT (β = 0.185, 95% CI [0.071, 0.299]); H1 is thus supported. CU significantly and positively predicted all three mediating variables: the standardized path coefficient for PCEB was 0.338 (95% CI [0.212, 0.454]), supporting H2; that for PBEB was 0.284 (95% CI [0.151, 0.410]), supporting H3; and that for PEEB was 0.383 (95% CI [0.254, 0.499]), supporting H4. Of the three mediating variables, both PCEB (β = 0.384, 95% CI [0.244, 0.516]) and PEEB (β = 0.346, 95% CI [0.187, 0.502]) were significantly positively associated with AT; however, the association between the third mediating variable, PBEB, and AT was not significant (β = −0.072, 95% CI [−0.206, 0.060]).

As shown in Table 7, the mediating effect analysis revealed that the indirect effect through PCEB was significant, with a standardized value of 0.100 (95% CI [0.053, 0.164]) and accounting for 28.8% of the total effect. The indirect effect through PEEB was also significant, with a standardized value of 0.102 (95% CI [0.050, 0.173]) and accounting for 29.7% of the total effect. H5 and H7 are thus supported. However, the indirect effect through PBEB was not significant, with a standardized value of −0.015 (95% CI [−0.052, 0.013]); the confidence interval included 0, and therefore H6 is not supported. The total effect of CU on AT was 0.335 (95% CI [0.231, 0.454]), of which the direct effect was 0.185, accounting for 55.2% of the total effect, while the total indirect effect was 0.188, accounting for 56.1% of the total effect. In conclusion, the indirect effects of CU on AT via PCEB and PEEB were statistically significant, indicating partial mediation in the statistical sense.

To examine whether the substantive conclusions were dependent on the choice of estimation method, the same model was re-estimated using maximum likelihood (ML) estimation with robust standard errors (MLR). The ML estimation converged successfully and demonstrated acceptable model fit: χ²(142) =371.70, RMSEA = 0.075 [0.066, 0.084], CFI = 0.925, SRMR = 0.059. The direction and significance of all core path coefficients were fully consistent with the Bayesian results. Specifically, PCEB→AT (0.375, p < .001), PEEB→AT (0.347, p < .001), and PBEB→AT (–0.051, p = .259), among others, aligned perfectly with the Bayesian posterior estimates. Thus, the study’s conclusions are robust and not an artifact of the Bayesian estimation method, demonstrating cross-methodological consistency.

## 6. Discussion

### 6.1. Theoretical contributions

This study confirmed that students’ in-class AI usage in routine contexts without teacher intervention can lead to comprehensive enhancement of learning engagement. Studies on the relationship between AI usage and learning engagement have mostly been conducted within contexts of specific instructional interventions [6,19,27,70]. Focusing on in-class AI usage and in-class learning engagement, this study demonstrates a positive association between routine in-class AI usage and perceived learning engagement benefits, consistent with the consensus viewpoint of existing studies. Moreover, AI usage not only enhances overall learning engagement, but also comprehensively improves students’ engagement during class. However, these effects were more pronounced for the cognitive and emotional aspects of learning engagement than for behavioral engagement. This finding offers insights for understanding the classroom ecology of the triad of teachers, students, and AI.

This study also revealed the mediating roles of perceived cognitive and emotional engagement benefits in the link between students’ in-class AI usage behavior and their attitudes toward AI usage. Most relevant studies to date have treated learning engagement as an outcome variable [4,7,20,36], whereas few have explored the mediating role of perceived engagement benefits in linking AI usage to other variables. This study thus investigated the mediating effect of these perceived benefits in the relationship between in-class AI usage behavior and attitudes toward AI usage and further identified perceived cognitive and emotional engagement benefits, two more implicit dimensions of engagement, as mediators. In-class AI usage positively promotes perceived cognitive engagement benefits through knowledge construction and stimulation of thinking [6,36,44], and students with high perceived cognitive benefits will then convert their cognitive benefits into positive attitudes toward AI usage. Meanwhile, in-class AI usage enhances emotional engagement through personalized feedback, interactivity, and entertainment [7,9,20,39,55]. Students with high perceived emotional benefits will associate the positive emotions they experience in class, such as learning pleasure and low anxiety, with AI [6,39,55], and this, in turn, evolves into positive attitudes toward AI usage. These two mediators, perceived cognitive and emotional engagement benefits, were found to make comparable contributions to the indirect effect, indicating that both the cognitive benefits and the emotional benefits of AI use are of great value. These findings provide important insights for understanding these cognitive and emotional psychological mechanisms in the context of in-class AI usage.

By contrast, this study found that perceived behavioral engagement benefits did not mediate the relationship between in-class AI usage behavior and attitudes. Existing studies have yielded evidence both for and against the claim that AI use enhances behavioral engagement. Most studies support a positive association [7,31,36,37], but some studies indicate that negative or complex relationships may exist, and thus it is necessary to consider the possible moderating roles of teaching context, task design, and teacher support [4,29,40]. Although this study confirmed that AI usage can enhance behavioral engagement, it also found that these perceived behavioral benefits did not mediate the relationship between AI usage behavior and attitude toward AI. This implies that the enhancement in behavioral engagement produced by AI use may not contribute to the formation of positive attitudes. Unlike cognitive and emotional engagement, behavioral engagement is a more explicit type of learning engagement, and the value of their enhancement is not as significant as that of the former two. In the context of Chinese higher education, students’ active speaking and in-depth interaction are generally insufficient in classroom settings due to the influence of teaching culture, assessment methods, and authority structure, and students in China are frequently characterized by passive participation and reticence in classroom discourse [13,71].

Consistent with the theoretical prediction that perceived behavioral engagement benefits should show the weakest mediating effect, the results revealed a non-significant indirect effect for perceived behavioral engagement benefits, while perceived cognitive and emotional engagement benefits showed significant positive indirect effects. Thus, the pattern of results partially supports the differentiated predictions, with the caveat that no evidence was found for behavioral engagement mediation.

### 6.2. Practical implications

The results of this study offer a number of practical implications as well. First, AI tools should be integrated into instructional design. In-class AI usage can significantly enhance students’ cognitive engagement (e.g., deep thinking, information integration) and emotional engagement (e.g., learning interest, self-confidence) and thereby facilitate the formation of positive attitudes. Teachers should take full advantage of these findings to optimize their classroom teaching design. AI-integrated tasks such as human-machine collaborative analysis and AI-assisted brainstorming can be designed to stimulate students’ cognitive and emotional engagement. Teachers should be encouraged to introduce AI-enhanced inquiry-based learning into their classes, to guide their students in conducting comparison and reflection. AI can also be used to provide personalized learning support, thereby enhancing students’ self-efficacy and learning motivation.

Guidance for classroom AI usage should be optimized. Given the prevalent use of AI in classroom learning, teachers should not simply prohibit AI, but instead should regard it as a pedagogical aid, incorporating it into course tasks and assessments to construct a teacher-student-AI classroom ecosystem that empowers classroom teaching. Teachers can provide guidance for students on appropriate AI use in classroom settings in their syllabi. When facilitating students’ AI usage, teachers should emphasize the cognitive challenge and emotional resonance of the task designs, and they should preclude the rote and mechanical use of AI tools. AI learning workshops should be offered to teach students how to leverage AI for advanced thinking tasks like in-depth analysis and creative expression.

A critical examination should be conducted on behavioral engagement in AI-supported classroom settings. The current study find that perceived behavioral engagement benefits cannot mediate the relationship between AI usage and attitudes toward AI, implying that superficial behavioral performance is insufficient to realize the educational value of AI. In teaching practice, AI should neither be used merely for the sake of doing so, nor should its usage be oversimplified, including such superficial practices as content generation and automated question answering [16]. Instead, emphasis should be placed on promoting deep learning and fostering emotional experiences.

### 6.3. Research limitations and future directions

This study has some limitations worth noting, as they point to directions for future research. First, the participants in the research sample were limited to students from a single research-intensive university in China, and the sample size (N = 287) was relatively small. This may limit the generalizability of the findings to students in other types of institutions or different cultural contexts. Researchers conducting future studies are recommended to extend the sampling scope and conduct comparative investigations with students across different countries.

Second, the present study drew its primary empirical evidence from questionnaire survey data, which is dependent on students’ self-reports. Although the survey was anonymous and no personally identifiable information was collected, the possibility of social desirability bias—where students might overreport ‘appropriate’ AI usage or engagement levels—cannot be fully excluded. Future research could incorporate qualitative research methodologies such as semi-structured interviews or systematic classroom observations to conduct a deeper exploration into the relationships among AI usage behaviors, attitudes, and learning engagement within classroom contexts.

Third, this study only focused on three common dimensions of learning engagement, without incorporating other dimensions such as agentic engagement. Agentic engagement is a relatively new dimension, which involves how students actively contribute to their own learning process [72]. Agentic engagement exerts a significant association with the classroom environment as well as on students’ intrinsic learning motivation and their academic attainment [73,74]. Future research could expand the scope to explore agentic engagement and its role in the context of in-class AI usage.

Fourth, the cross-sectional design prevents causal conclusions about the mediation effects. Reverse causation (e.g., positive attitudes→more AI usage) remains possible. Thus, all findings are correlational. Longitudinal or experimental designs are needed to establish causality.

Fifth, the mediating constructs in this study measure students’ perceived benefits of AI use regarding their engagement (PCEB, PBEB, PEEB), rather than engagement metrics. Since both the mediators and the outcome variable (attitudes) are self-report measures that reference AI and involve evaluative judgments, the observed indirect effects may be partly driven by construct overlap. Although our discriminant validity analysis confirmed the statistical distinctness of these constructs, future research would benefit from incorporating objective measures of engagement.

In addition, the Attitudes scale was newly developed for this study and, although it showed strong psychometric properties (α = 0.884; factor loadings>0.82), it lacks prior validation. Future research should replicate our findings using established measures.

Furthermore, the In-Class AI Usage scale measured the general frequency of AI tool utilization for various tasks, but it did not distinguish between specific types of AI (e.g., generative AI for content creation versus search-based AI for fact-checking). Given that different types of AI applications likely exert varying effects on specific dimensions of learning engagement, future studies should develop more granular instruments to capture these distinct usage patterns.

Finally, the changes in and associations of students’ behavioral engagement in the context of in-class AI usage are important issues worthy of further exploration. Unlike the perceived cognitive and emotional engagement benefits, perceived behavioral engagement benefits showed no significant indirect effect on attitudes. This null finding suggests that, in the current sample, perceived behavioral engagement benefits did not function as a mediator in the relationship between AI usage and attitudes. Future research with alternative measures or designs may further explore the role of behavioral engagement in this process.

## 7. Conclusions

This study investigates the relationship between students’ routine, in-class AI usage behavior and their attitudes toward it, as well as the mediating role of learning engagement. The results indicate that in-class AI usage is directly associated with students’ attitudes, and is also indirectly associated with attitudes through the mediators of perceived cognitive and emotional engagement benefits, both of which exert comparable mediating effects. In-class AI usage has become prevalent among university students, who generally hold positive attitudes toward it; moreover, it can contribute to comprehensive enhancement in their learning engagement. The value of AI in classrooms lies not only in improving learning efficiency, but also in reshaping students’ learning experiences by enhancing their cognitive and emotional engagement. Educational practice should move beyond the debate over whether AI use should be permitted, and instead the focus should be shifted to designing human-AI collaborative learning experiences that use AI as a catalyst for fostering deep learning and student development. This study recommends that educators integrate AI tools into their teaching design, optimize their guidance on in-class AI usage, and look beyond superficial behavioral participation to focus primarily on facilitating deep cognitive integration and fostering positive emotional experiences for their students. One key finding of this study that warrants special attention is that, although in-class AI usage can boost all dimensions of learning engagement, the potential link between perceived behavioral engagement benefits and positive attitudes toward in-class AI usage remains unconfirmed. In contrast to perceived cognitive and emotional engagement benefits, perceived behavioral engagement benefits showed no significant indirect effect on attitudes. This null finding warrants further investigation in subsequent research.

## Supporting information

S1 File Minimal data set. The excel file contains the raw data used for the statistical analysis of learning engagement, including in-class AI usage and student perceptions.
(XLSX)

S2 File Appendix. The document lists the detailed measurement items for Perceived cognitive engagement benefits(PCEB), Perceived behavioral engagement benefits (PBEB), and Perceived emotional engagement benefits (PEEB).
(DOCX)
