# Supplementary material for: In-class AI usage and university students’ attitudes: The mediating role of perceived learning engagement benefits
Source: PLoS One. 2026 Aug 4;21(8):e0355490. doi: 10.1371/journal.pone.0355490 (PMC13436728; doi:10.1371/journal.pone.0355490)
Supplement: Supplementary file 1 [file pone.0355490.s002.docx]

**Appendix A**

**Abbreviations**

The following abbreviations are used in this manuscript:

CU	In-Class AI Usage

PCEB Perceived Cognitive Engagement Benefits

PBEB Perceived Behavioral Engagement Benefits

PEEB Perceived Emotional Engagement Benefits

AT	Attitudes toward In-Class AI Usage

**Items of the Perceived Cognitive Engagement Benefits (PCEB)**

Using AI during class helps me understand complex concepts more efficiently.

Using AI during class encourages me to think more deeply about the course con-tent.

Using AI during class enables me to access a wider range of extended knowledge.

Using AI during class helps me develop more diverse perspectives on learning content.

**Items of the Perceived Behavioral Engagement Benefits (PBEB)**

Using AI during class makes me more willing to actively respond to the instructor’s questions.

Using AI during class increases my participation in discussions and interactions with classmates.

Using AI during class enhances my active involvement in classroom activities.

**Items of the Perceived Emotional Engagement Benefits (PEEB)**

Using AI during class increases my interest in the course content.

Using AI during class enhances my belief that I am capable of learning the mate-rial.

Using AI during class makes me feel more confident when dealing with chal-lenging learning tasks.

Using AI during class provides me with a stronger sense of learning accomplish-ment.
