# Supplementary material for: In-class AI usage and university students’ attitudes: The mediating role of perceived learning engagement benefits
Source: PLoS One. 2026 Aug 4;21(8):e0355490. doi: 10.1371/journal.pone.0355490 (PMC13436728; doi:10.1371/journal.pone.0355490)
Supplement: Supplementary file 2 [file pone.0355490.s004.docx]

**Response to Reviewers**

**Dear Editors and Reviewers,**

We would like to express our sincere gratitude to the Academic Editor and the Reviewer for their rigorous evaluation of our manuscript (PONE-D-26-08042) and their highly constructive feedback. We have carefully considered all the comments and have revised the manuscript accordingly. We believe that these revisions have substantially improved the theoretical foundation, methodological rigor, and clarity of our paper.

Below, we provide a point-by-point response to each comment. The original comments are presented in bold, followed by our responses and the corresponding modifications made in the manuscript.

**Part I: Responses to Journal Requirements (Academic Editor)**

**1. Please ensure that your manuscript meets PLOS ONE's style requirements, including those for file naming.**

- **Response:** Thank you for this reminder. We have carefully checked the revised submission package and ensured that the manuscript has been revised to comply with PLOS ONE’s style requirements, including the formatting of the manuscript and the naming of the uploaded files. We will submit the revised files using the journal’s required file labels and formats, including “Response to Reviewers,” “Revised Manuscript with Track Changes,” and “Manuscript.”

**2. Please include your full ethics statement in the ‘Methods’ section of your manuscript file. In your statement, please include the full name of the IRB or ethics committee who approved or waived your study, as well as whether or not you obtained informed written or verbal consent. If consent was waived for your study, please include this information in your statement as well.**

- **Response:** Thank you for the reminder. We have incorporated the full ethics statement into the 'Methods' section as requested. Specifically, the details regarding the ethical waiver from the Ethics Committee of Beihang University and the informed written consent procedures have been added to the end of Section 4.1 (Survey samples).

**3. Thank you for stating the following financial disclosure:**

**This research was supported by the Fundamental Research Funds for the Central Universities, Key Teaching Reform Project of Beihang University, and Research and Development Fund Project for Postgraduate Education of Beihang University (JG2024030).**

**Please state what role the funders took in the study. If the funders had no role, please state: "The funders had no role in study design, data collection and analysis, decision to publish, or preparation of the manuscript."**

**If this statement is not correct you must amend it as needed.**

**Please include this amended Role of Funder statement in your cover letter; we will change the online submission form on your behalf.**

- **Response:** Thank you for your guidance. The funders had no role in study design, data collection and analysis, decision to publish, or preparation of the manuscript. We have added this required statement to the "Funding" section at the end of the revised manuscript. Additionally, we have explicitly included this amended Role of Funder statement in the new cover letter accompanying this revised submission. Please kindly review it.

**4. Please upload a copy of Figure 1, 2, to which you refer in your text on page 10, 15. If the figure is no longer to be included as part of the submission please remove all reference to it within the text.**

- **Response:** Thank you for this reminder. We apologize for the confusion caused by the initial file naming. Following PLOS ONE’s guidelines, we have submitted the figures as separate files rather than embedding the images in the manuscript. The files previously labeled as "S1_Fig.tif" and "S2_Fig.tif" are indeed the "Figure 1" and "Figure 2" referred to in the text. To ensure clarity and consistency, we have now renamed these image files to Fig1.tif and Fig2.tif and re-uploaded them to the submission system as "Figure" items.

**5. Please include a separate caption for each figure in your manuscript.**

- **Response:** Thank you for this reminder. In accordance with the journal's formatting guidelines, a separate caption has been added for each figure in the revised manuscript. Specifically, we have moved the captions from the end of the manuscript directly into the main text, inserting them immediately after their first citation (in Section 3.4 and Section 5.4, respectively), and deleted the "(TIF)" text at the end of the captions. Please kindly review.

**6. If the reviewer comments include a recommendation to cite specific previously published works, please review and evaluate these publications to determine whether they are relevant and should be cited. There is no requirement to cite these works unless the editor has indicated otherwise.**

- **Response:** Thank you for your guidance. In our case, the reviewers did not recommend any specific publications for citation.

**Part II: Responses to Reviewer #1**

**A. Responses to Specific Comments**

**1. The title is too long and overly descriptive, reducing clarity and impact. Phrase “Are there differences across dimensions of engagement?” appears exploratory rather than theory-driven.**

- **Response:** Thank you very much for this helpful comment. We agree that the original title is somewhat lengthy and that the interrogative phrase at the end may make the study appear more exploratory than theoretically focused. To improve clarity and concision, we have revised the title. **Revised title:** “*I****n-class AI usage and university students' attitudes: The mediating role of learning engagement***”

**2. Convenience sampling from a single research-intensive university (H University) in China severely limits generalizability. The sample (N=287) is relatively small for the complexity of the structural equation model tested.**

- **Response:** We acknowledge the limitations regarding sampling and sample size. Using convenience sampling at a single institution indeed restricts the generalizability of our results. We have revised the "6.3 Research limitations and future directions" section to reflect this more accurately. **Modification:** In Section 6.3, we explicitly stated: "First, the participants in the research sample were limited to students from a single research-intensive university in China, and the sample size (N=287) was relatively small. This may limit the generalizability of the findings to students in other types of institutions or different cultural contexts."

**3. The sample disproportionately represents science and engineering students (202 vs. 85 humanities/social sciences), yet no analysis examines whether findings differ by discipline. This is a significant omission given that AI usage patterns likely vary substantially across fields.**

- **Response:** Thank you for your comment. Your insight is very reasonable—AI usage patterns may indeed differ among students from different academic disciplines. We conducted differential analyses on five core variables across disciplinary fields and found that three showed no significant differences, while two did show significant differences, while both with small effect sizes. This indicates that although the disciplinary differences are statistically significant, their practical impact is very small. Due to the unbalanced sample composition (overrepresentation of science and engineering students), we did not conduct direct group comparisons. (Added to Section 5.3)

**4. No power analysis is reported to justify that N=287 is sufficient for the SEM with multiple mediators and Bayesian estimation.**

●	**Response:** Thank you for this important methodological point. We would like to clarify that traditional frequentist power analysis is not directly applicable to Bayesian estimation, because Bayesian inference does not rely on null hypothesis significance testing or the concept of statistical power in the Neyman–Pearson sense. Instead, the adequacy of sample size in Bayesian SEM is evaluated through MCMC convergence diagnostics and the precision of posterior estimates.

In our study, with N = 287 and 82 free parameters, the following evidence supports that the sample size is sufficient for stable estimation:

Convergence: All MCMC chains converged well, with PSR (Potential Scale Reduction Factor) ≤ 1.001, well below the recommended threshold of 1.05. Trace plots and autocorrelation plots showed no abnormal trends.

Effective Parameters: The pD (effective number of parameters) was 81.6, which is very close to the total number of free parameters (82), indicating that nearly all parameters were identified by the data rather than solely influenced by priors.

Estimation Precision: The 95% credible intervals for all parameters were reasonably narrow, providing sufficient precision for substantive interpretation.

Thus, while traditional power analysis was not performed, the reported Bayesian convergence diagnostics (added to Section 5.4) fully justify that N = 287 is adequate for the current model complexity.

**5. All variables (CU, CE, BE, EE, AT) were measured using self-report questionnaires from the same respondents at a single time point. This introduces substantial risk of common method variance, which the authors do not adequately address. The Harman's single-factor test or more rigorous approaches (e.g., marker variable technique) are absent.**

- **Response:** Thank you for this important methodological observation. In this study, Harman's single-factor test was used to examine common method bias. An unrotated principal component analysis on all measurement items revealed five factors with eigenvalues greater than 1.0, with the first factor accounting for 38.114% of the total variance, which is below the recommended threshold of 50% (Podsakoff et al., 2003). This indicates that common method bias is not a serious concern in this study and is unlikely to substantially distort the relationships among the variables. (Added to Section 4.3)

**6. The study uses cross-sectional data but makes causal claims about mediation (e.g., "AI usage affected students' attitudes both directly and indirectly"). Without longitudinal or experimental design, the direction of causality is ambiguous. Reverse causation (positive attitudes → more AI usage) is equally plausible.**

- **Response:** Thank you for this critical methodological observation. The reviewer is correct that cross-sectional data cannot support strong causal claims about mediation. The original wording has been revised throughout the manuscript to avoid causal language. The following actions have been taken:

1. Revision of causal language. All instances of causal claims (e.g., "affected," "influenced," "impacted") have been replaced with non-causal, associative language appropriate for cross-sectional data (e.g., "was associated with," "predicted", "was related to,"). The abstract, results, and discussion sections have all been revised accordingly.
2. Limitations statement. A clear limitation has been added to the Discussion section (Section 6.3) explicitly stating that the cross-sectional design precludes causal inference and that longitudinal or experimental designs are needed to establish temporal precedence and causality.

**7. The Attitudes scale (3 items) was "compiled independently by the researchers" with no reported validation beyond this study. The authors should provide evidence of prior validation or at minimum report item wording and pilot testing results.**

- **Response:** We appreciate the reviewer's vigilance. The three attitude items were reviewed by three subject matter experts. The complete wording of the three attitude items is provided in the Appendix (S2 File). In this study, the Cronbach's α for the Attitudes scale was 0.884. Exploratory factor analysis (EFA) extracted a single factor with an eigenvalue of 2.443, accounting for 81.431% of the total variance. The standardized factor loadings of the three items ranged from 0.890 to 0.920, with no evidence of double loading. Together, these indices consistently demonstrate that this newly developed Attitudes scale has good reliability and validity, indicating that the instrument is suitable for use in subsequent research (Added to Section 4.2). Nevertheless, we acknowledge the lack of prior validation as a limitation and have noted this in the Limitations section (Section 6.3) of the revised manuscript.

**8. The In-Class AI Usage Behavior Scale asks about frequency ("Never" to "Very frequently") but does not distinguish types of AI use (e.g., generative AI for content creation vs. search-based AI for fact-checking). Different AI uses likely have different effects on engagement dimensions.**

- **Response:** We sincerely thank the reviewer for pointing out this important nuance. We agree that treating AI usage primarily as a frequency variable without differentiating the specific types of AI tools (e.g., generative vs. search-based) is a limitation of our measurement instrument, as different AI functionalities may distinctively impact learning engagement. To address this, we have added this point to the research limitations (Section 6.3) as a critical direction for future studies.

**9. Social desirability bias is unaddressed. Students may overreport "appropriate" AI usage or engagement levels.**

- **Response:** Thank you for this helpful comment. We agree that social desirability bias is a potential concern in questionnaire-based research of this kind, and that students may have a tendency to overreport their AI usage and engagement when responding to related questions. In the present study, the survey was conducted anonymously, participation was voluntary, and no personally identifiable information was collected, which may help reduce respondents’ pressure to provide socially desirable answers. To address this concern more explicitly, we have added a brief statement in the limitations section (Section 6.3).

**10. The paper invokes Self-Perception Theory (Bem, 1972), Cognitive Consistency Theory, and Affective Events Theory but does not integrate them into a coherent framework. How do these theories work together? Which predictions do they make about differential mediation across engagement dimensions?**

- **Response:** Thank you for this insightful comment. We agree that the original manuscript did not sufficiently integrate the three theoretical perspectives. We have now added an integrated framework ("three-layer progressive consistency framework") of the three perspectives in the research model section (Section 3.4) to clarify how these theories work together and predict the mediating effects of different engagement dimensions based on these theories.

**11. The authors claim Bayesian analysis is "more suitable for relatively small sample sizes" but provide no comparison with frequentist approaches or sensitivity analyses. The choice of priors is not specified, which is a fundamental omission in Bayesian work.**

- **Response:** We thank the reviewer for these important points. We apologize for this omission. In the revised manuscript (Section 4.3), we have added a detailed Prior Specification subsection. Bayesian estimation employed Mplus default diffuse priors: factor loadings, path coefficients, and intercepts followed a normal distribution with a mean of 0 and a variance of 10¹⁰, while residual variances and covariances were assigned non-informative inverse-gamma priors. To examine the sensitivity of posterior inference to prior specifications, we re-estimated the model by replacing the priors for all structural path coefficients with weakly informative normal priors N(0,1), keeping all other specifications unchanged. The results showed that the Bayesian inference in this study is robust and not sensitive to the choice of priors. Following the reviewer's suggestion, we also estimated the same model using maximum likelihood (ML) estimation as a comparative approach (added to Section 5.4). Critically, the direction and significance of the core paths were entirely consistent with the Bayesian results. This demonstrates that the conclusions of this study are robust and consistent across two different estimation approaches (Bayesian and frequentist ML), providing strong evidence for the cross-methodological robustness of our findings.

**12. The path from BE → AT (β = -0.074, CI [-0.205, 0.056]) is non-significant, yet the authors interpret this as "behavioral engagement operates through a mechanism distinct from that of cognitive and emotional engagement." This is over-interpretation of a null finding. The confidence interval includes zero; the correct conclusion is that no evidence was found for mediation, not that a distinct mechanism exists.**

- **Response:** We thank the reviewer for this critical methodological correction. The reviewer is absolutely correct. We acknowledge that our original interpretation of the non-significant BE → AT path was inappropriate and constituted over-interpretation of a null finding. We agree that a confidence interval that includes zero (CI [-0.205, 0.056]) provides no evidence for a mediation effect. Interpreting this null result as evidence of a "distinct mechanism" is statistically unjustified. We have removed this incorrect interpretation from the manuscript. The correct conclusion from this finding is simply that no significant evidence was found for behavioral engagement as a mediator in the relationship between AI usage and attitudes. We have revised the relevant sections of the manuscript (Abstract, Section 6.1, 6.3, and Conclusions) accordingly.

**13. No information is provided about missing data. Were there incomplete responses? How were they handled?**

- **Response:** Thank you for this comment. No missing data were present in the final dataset. After screening, all 287 included cases had complete responses on all scale items. This information has been added to the 4.3 Data analysis section: "All 287 valid questionnaires contained complete responses, with no missing data on any scale item."

**B. Responses to Standardized Questions**

**1.Is the manuscript technically sound, and do the data support the conclusions? The manuscript must describe a technically sound piece of scientific research with data that supports the conclusions. Experiments must have been conducted rigorously, with appropriate controls, replication, and sample sizes. The conclusions must be drawn appropriately based on the data presented.**

**Reviewer #1: Partly**

- **Response:** Thank you for your feedback. We have made revisions and provided responses following the specific reviewer comments (3, 4, 5, 6, 11). After revising the manuscript based on these comments, I believe the paper is more technically sound, and the data support the conclusions. We would like to express our sincere gratitude again to the reviewers for their valuable feedback.

**2.Has the statistical analysis been performed appropriately and rigorously?**

**Reviewer #1: Yes**

- **Response:** Thank you for confirming that the statistical analysis has been performed appropriately and rigorously. We truly appreciate your positive evaluation and valuable acknowledgment.

**3.Have the authors made all data underlying the findings in their manuscript fully available?**

**The PLOS Data policy requires authors to make all data underlying the findings described in their manuscript fully available without restriction, with rare exception (please refer to the Data Availability Statement in the manuscript PDF file). The data should be provided as part of the manuscript or its supporting information, or deposited to a public repository. For example, in addition to summary statistics, the data points behind means, medians and variance measures should be available. If there are restrictions on publicly sharing data—e.g. participant privacy or use of data from a third party—those must be specified.**

**Reviewer #1: No**

- **Response:** Thank you for your guidance. The means of the five core variables have been added to the data file. If any additional data needs to be included, please let us know.

**4.Is the manuscript presented in an intelligible fashion and written in standard English?**

**PLOS ONE does not copyedit accepted manuscripts, so the language in submitted articles must be clear, correct, and unambiguous. Any typographical or grammatical errors should be corrected at revision, so please note any specific errors here.**

**Reviewer #1: Yes**

- **Response:** Thank you for confirming that the manuscript is intelligible and written in standard English. We greatly appreciate your positive evaluation. We will continue to ensure that the text remains clear, coherent, and grammatically correct.

We hope that these revisions fully address your concerns and meet the high standards of PLOS ONE. Thank you once again for your constructive guidance.

**Sincerely,**

**The Authors**
