# Supplementary material for: In-class AI usage and university students’ attitudes: The mediating role of perceived learning engagement benefits
Source: PLoS One. 2026 Aug 4;21(8):e0355490. doi: 10.1371/journal.pone.0355490 (PMC13436728; doi:10.1371/journal.pone.0355490)
Supplement: Supplementary file 3 [file pone.0355490.s005.docx]

**Response to Reviewers**

**Dear Editors and Reviewers,**

We would like to express our sincere gratitude to you for your rigorous evaluation of our manuscript (PONE-D-26-08042) and your highly constructive feedback. We have carefully considered all the comments and have revised the manuscript accordingly. We believe that these revisions have substantially improved the theoretical foundation, methodological rigor, and clarity of our paper.

Additionally, before proceeding to the point-by-point responses, we would like to briefly note that a “Declaration on Data Reuse" has been included at the very end of this document to clarify the relationship between this study and a recently published related work.

Below, we provide the point-by-point response to each comment. The original comments are followed by our responses and the corresponding modifications, which are highlighted in the revised manuscript.

**Reviewer #1:
Comment 1.** **The authors have fully met the requirements of the reviewer comments. The manuscript is ready for acceptance from a review-response perspective.**

- **Response:**

We sincerely thank the reviewer for the positive and encouraging assessment of our revisions. We are very glad to hear that our point-by-point responses have fully addressed all the concerns raised. We truly appreciate the reviewer’s time and constructive suggestions throughout this process, which have significantly improved the quality of our manuscript.

**Reviewer #3:
Comment 1. The mediators measure perceived benefit not engagement. This is my key point. The instruments do not measure engagement. They measure agreement that AI enhances engagement. The authors say CE is "perception of whether in-class AI usage enhances cognitive engagement." Same for BE and EE. So these are evaluative. AI-referential. They are close to perceived usefulness. The authors invoke TAM and perceived usefulness themselves on the mediator→attitude paths. All same-method and highly overlapping constructs "I think AI boosts my thinking" predicting "I like AI" ishould be described more carefully.**

**Suggestion: relabel the constructs accurately throughout. They are perceived engagement benefits. Not engagement. and discuss how much indirect effect might be construct overlap rather than mechanism.**

- **Response:**

We sincerely thank the reviewer for this crucial insight. We completely agree that our instruments measure the students' evaluative agreement that AI enhances their engagement, rather than objective engagement metrics. To accurately reflect this, we have systematically relabeled the constructs throughout the entire manuscript—including the title, abstract, hypotheses, instruments, and discussion—to "perceived cognitive engagement benefits (PCEB)", "perceived behavioral engagement benefits (PBEB)", and "perceived emotional engagement benefits (PEEB)".

Furthermore, we have followed your suggestion to explicitly discuss the issue of construct overlap. We added a new paragraph to the limitations section to address how the indirect effects might be partially attributable to this overlap or a generalized positive appraisal rather than a purely sequential mechanism.

- **Modification:**

(1) To ensure conceptual consistency from the very beginning, we have modified the title of the manuscript to replace the general term of learning engagement with our specific measured variable.

**Modified title:** In-class AI usage and university students' attitudes: The mediating role of perceived learning engagement benefits

(2) In **Section 6.3 (Research limitations and future directions)**, we have added a new fifth paragraph between the fourth limitation and the subsequent discussion to explicitly acknowledge the measurement boundaries and address the potential issue of construct overlap.

Section 6.3 (Research limitations and future directions):

Fifth, the mediating constructs in this study measure students' perceived benefits of AI use regarding their engagement (PCEB, PBEB, PEEB), rather than engagement metrics. Since both the mediators and the outcome variable (attitudes) are self-report measures that reference AI and involve evaluative judgments, the observed indirect effects may be partly driven by construct overlap. Although our discriminant validity analysis confirmed the statistical distinctness of these constructs, future research would benefit from incorporating objective measures of engagement. **(Section 6.3)**

**Comment 2. The revision adds a "three-layer progressive consistency framework." suggesting that it is developmental when in fact t is parallel. It is cross-sectional. Three mediators side by side. It cannot test temporal order. So the framework's core claims are should be more tentatively claimed. Either scale the theory back to what the model evaluates. Or state clearly that the framework is an interpretive heuristic. Not a tested structure.**

- **Response:**

We greatly appreciate your rigorous evaluation of our theoretical framework and empirical model. You are correct that because our survey data is cross-sectional and the three mediators are evaluated in a parallel configuration, we cannot empirically verify a temporal order. We agree that our previous claims regarding a "developmental" or "progressive" trajectory were overstated. To address this, we have tempered our theoretical claims in Section 3.4. We revised the language to present a "three-dimensional theoretical framework" with "complementary interpretive angles" rather than a sequential or developmental process. Most importantly, we have added a clear statement explicitly defining the framework as an interpretive heuristic rather than a tested structure.

- **Modification:**

**Section 3.4 (Research model):**

It is important to note that, while this framework proposes multiple layers of psychological processing, the cross-sectional survey design does not allow for empirical tests of temporal sequence or causal direction among the dimensions. Accordingly, this theoretical framework should therefore be interpreted as a conceptual heuristic to map concurrent psychological channels rather than a temporally verified path structure. **(Section 3.4)**

**Comment 3. The "proportion mediated" figures (53.6% / 54.9%) are unstable with small effects. De-emphasize them.**

- **Response:**

We sincerely thank the reviewer for this valuable methodological advice. We fully agree that the "proportion mediated" statistic tends to be unstable when the indirect effect is relatively small.

Accordingly, we have removed the unstable figures. We have completely deleted all reports of the "proportion mediated" values (53.6% and 54.9%) from the revised manuscript.

- **Modification:**

The following sentence was deleted from Section 5.4 (Hypothesis testing):

"Of the significant indirect effects, CE and EE showed comparable mediating effects, accounting for 53.6% (95% CI [32.8%, 75.6%]) and 54.9% (95% CI [30.3%, 81.8%]) of the total indirect effects, respectively." (Deleted)

**Comment 4. One recommendation contradicts a finding. The paper says use AI to "stimulate behavioral engagement." But BE does not relate to attitudes. Reconcile this.**

- **Response:**

We sincerely appreciate the reviewer’s astute observation regarding this logical contradiction. You are correct that advising educators to "stimulate behavioral engagement" directly conflicted with our empirical finding that perceived behavioral engagement benefits do not significantly relate to or mediate student attitudes.

To resolve this contradiction and ensure strict alignment between our empirical evidence and pedagogical recommendations, we have systematically removed and rephrased the active advocacy for stimulating behavioral engagement in both the Practical Implications and Conclusions sections.

- **Modification:**

(1) In **Section 6.2 (Practical implications)**, the third paragraph has been revised as follows:

A critical examination should be conducted on behavioral engagement in AI-supported classroom settings. The current study find that perceived behavioral engagement benefits cannot mediate the relationship between AI usage and attitudes toward AI, implying that superficial behavioral performance is insufficient to realize the educational value of AI. In teaching practice, AI should neither be used merely for the sake of doing so, nor should its usage be oversimplified, including such superficial practices as content generation and automated question answering [16]. Instead, emphasis should be placed on promoting deep learning and fostering emotional experiences. **(Section 6.2)**

(2) In **Section 7 (Conclusions),** the relevant pedagogical recommendation paragraph has been amended as follows:

Educational practice should move beyond the debate over whether AI use should be permitted, and instead the focus should be shifted to designing human-AI collaborative learning experiences that use AI as a catalyst for fostering deep learning and student development. This study recommends that educators integrate AI tools into their teaching design, optimize their guidance on in-class AI usage, and look beyond superficial behavioral participation to focus primarily on facilitating deep cognitive integration and fostering positive emotional experiences for their students. **(Section 7)**

**Comment 5. Control variables (gender, grade, major) are not fully specified in the structural model. This matters. The disciplinary differences for CE and BE were significant.**

- **Response:**

We sincerely thank the reviewer for identifying this critical issue. In response, we have made the following revisions:

We re-ran the structural equation model analysis. In the final adopted model, gender, grade, and major were specified as covariates predicting all endogenous variables (i.e., the independent variable, mediator, and dependent variable) in the structural model.

We compared the hypothesis testing results between the two models and confirmed that all hypothesized paths remain significant with unchanged directions before and after covariate adjustment (see the comparison below). This demonstrates that our findings are robust and not contingent upon whether covariates are controlled. Importantly, after controlling for major as a covariate, all our hypothesized relationships continue to hold, indicating that the observed effects are generalizable across disciplines.

- **Modification:**

In **Section 5.4 (Hypothesis testing)**, we have updated the model description to explicitly clarify the inclusion of covariates, and the relevant paragraph has been revised as follows:

In this study, the hypotheses were tested using SEM conducted in Mplus 8.3. Bayesian analysis was employed with the Markov Chain Monte Carlo (MCMC) method for parameter estimation. The model employed two parallel chains over 40,000 iterations, with the first 10,000 iterations used as burn-in. Posterior samples were saved at intervals of every 10 iterations. Convergence diagnostics indicated that the final value of the potential scale reduction factor (PSRF) was 1.009, and the PSR values for all parameters were close to 1.000. This is well below the strict threshold of 1.05, thus verifying the reliability of the subsequent parameter estimation results [68, 69]. In addition, examination of the trace plots and autocorrelation plots for the parameters’ posterior distributions revealed no abnormal trends or high autocorrelation, further verifying the good model convergence. All analyses controlled for gender, grade, and major as covariates affecting the independent, mediating, and dependent variables. **(Section 5.4)**

Furthermore, the data in the corresponding tables and the path diagram have been updated to reflect the newly estimated coefficients after covariate adjustment:

**1. Comparison of Table 6 (Before and After Modification)**

**Original Table 6: Standardized path coefficients of the SEM**

| Paths | Coefficient estimates | S.E. | Lower 95%CI | Upper 95%CI |
| --- | --- | --- | --- | --- |
| CU→AT | 0.197 | 0.058 | 0.083 | 0.308 |
| CU→CE | 0.338 | 0.061 | 0.212 | 0.453 |
| CU→BE | 0.272 | 0.066 | 0.139 | 0.397 |
| CU→EE | 0.374 | 0.061 | 0.249 | 0.489 |
| CE→AT | 0.380 | 0.068 | 0.242 | 0.509 |
| BE→AT | -0.074 | 0.067 | -0.205 | 0.056 |
| EE→AT | 0.351 | 0.080 | 0.191 | 0.508 |

**Revised Table 6: Standardized path coefficients of the SEM**

| Paths | Coefficient estimates | S.E. | Lower 95%CI | Upper 95%CI |
| --- | --- | --- | --- | --- |
| CU→AT | 0.185 | 0.058 | 0.071 | 0.299 |
| CU→PCEB | 0.338 | 0.062 | 0.212 | 0.454 |
| CU→PBEB | 0.284 | 0.066 | 0.151 | 0.410 |
| CU→PEEB | 0.383 | 0.062 | 0.254 | 0.499 |
| PCEB→AT | 0.384 | 0.069 | 0.244 | 0.516 |
| PBEB→AT | -0.072 | 0.068 | -0.206 | 0.060 |
| PEEB→AT | 0.346 | 0.080 | 0.187 | 0.502 |

**2. Comparison of Table 7 (Before and After Modification)**

**Original Table 7: Results of multiple mediating effect test (standardized)**

| Standardized effects | Effect size proportion | SE | Estimates | 95% CI | |
| --- | --- | --- | --- | --- | --- |
|  |  |  |  | Lower Limit | Higher Limit |
| Standardized total effects |  |  |  |  |  |
| CU→AT | 100% | 0.058 | 0.347 | 0.241 | 0.469 |
| Standardized direct effects |  |  |  |  |  |
| CU→AT | 56.8% | 0.058 | 0.197 | 0.083 | 0.308 |
| Standardized indirect effects |  |  |  |  |  |
| CU→CE→AT | 28.8% | 0.028 | 0.100 | 0.053 | 0.164 |
| CU→BE→AT | -4.3% | 0.016 | -0.015 | -0.052 | 0.012 |
| CU→EE→AT | 29.7% | 0.032 | 0.103 | 0.050 | 0.174 |
| Total indirect effects | 54.5% | 0.039 | 0.189 | 0.120 | 0.273 |

**Revised Table 7: Results of multiple mediating effect test (standardized)**

| Standardized effects | Effect size proportion | SE | Estimates | 95% CI | |
| --- | --- | --- | --- | --- | --- |
|  |  |  |  | Lower Limit | Higher Limit |
| Standardized total effects |  |  |  |  |  |
| CU→AT | 100% | 0.057 | 0.335 | 0.231 | 0.454 |
| Standardized direct effects |  |  |  |  |  |
| CU→AT | 55.2% | 0.058 | 0.185 | 0.071 | 0.299 |
| Standardized indirect effects |  |  |  |  |  |
| CU→PCEB→AT | 53.8% | 0.029 | 0.100 | 0.053 | 0.164 |
| CU→PBEB→AT | - | 0.017 | -0.015 | -0.052 | 0.013 |
| CU→PEEB→AT | 54.8% | 0.032 | 0.102 | 0.050 | 0.173 |
| Total indirect effects | 56.1% | 0.039 | 0.188 | 0.119 | 0.273 |

**3. Comparison of Path Diagram (Before and After Modification)**

**Original Fig 2: Mediating path diagram. (Before controlling for covariates)**

**Revised Fig 2: Mediating path diagram. (After controlling for covariates)**

**Journal Requirements:**

**1.** **If the reviewer comments include a recommendation to cite specific previously published works, please review and evaluate these publications to determine whether they are relevant and should be cited. There is no requirement to cite these works unless the editor has indicated otherwise.**

- **Response:**

Thank you for the reminder. We confirm that no citations were recommended by the reviewers.

**2.** **Please review your reference list to ensure that it is complete and correct. If you have** **cited papers that have been retracted, please include the rationale for doing so in the manuscript text, or remove these references and replace them with relevant current references. Any changes to the reference list should be mentioned in the rebuttal letter that accompanies your revised manuscript. If you need to cite a retracted article, indicate the article’s retracted status in the References list and also include a citation and full reference for the retraction notice.**

- **Response:**

We thank the editor for this important reminder. Our responses are as follows:

(1) Retracted publications: We have carefully verified every entry in our reference list and confirm that none of the cited papers have been retracted. Therefore, no rationale for citing retracted articles is needed.

(2) Changes to the reference list: One replacement has been made. The original reference [61] has been substituted with a new publication. Details are provided below:

Removed (original [61]): Liu E, Dewaele JM, Wang J. Developing a short language classroom engagement scale (LCES) and linking it with needs satisfaction and achievement. System. 2024;120(1):103189. doi: 10.1016/j.system.2023.103189.

Added (new [61]): Fan W, Cheng L, Wang Y, Zhao Q, Li Y. In-class AI use and attitudes among university students: The different mediating roles of cognitive relief and cognitive offloading. Behav Sci. 2026;16(6):1014. doi: 10.3390/bs16061014.

The corresponding in-text citation has been updated accordingly, and all changes have been clearly marked in the revised manuscript for the editor's and reviewers' convenience.

**Declaration on Data Reuse**

In addition to addressing the reviewers' comments, we would also like to make a declaration regarding data reuse:

Data for the present study and Fan et al. (2026) (Available from: <https://www.mdpi.com/2076-328X/16/6/1014>） were sourced from the same large-scale survey project. The two investigations overlap on two constructs: in-class AI usage and user attitudes. Whereas Fan et al. (2026) addressed separate research questions focused on the mediating effects of cognitive relief and cognitive offloading. The descriptive statistics and reliability coefficients for these overlapping variables match exactly the values documented in Fan et al. (2026). These statistics are therefore reproduced in Tables 1–5 with proper referencing to facilitate readers’review. Corresponding citations to Fan et al. (2026) have also been supplemented in Section 4.2 (Instruments). Additionally, we have removed the specific measurement items for the two variables, CU (in-class AI usage) and AT (attitudes), from the Appendix.

We categorize this practice as legitimate data reuse. Despite sharing the aforementioned two measured variables, the two manuscripts diverge markedly in analytical frameworks, research objectives, and theoretical underpinnings, ultimately yielding entirely different empirical findings.

We sincerely apologize for any inconvenience caused by revisions related to this issue, and we highly appreciate the opportunity to publish our manuscript in this esteemed journal.

**Modifications regarding data reuse:**

In **Section 4.1 (Survey samples)**, we have added the following clarifying statement:

The data for this study were derived from a larger survey project reported in Fan et al. [61], which uses the same dataset but investigates distinct research questions concerning the mediating roles of cognitive relief and cognitive offloading. As such, the descriptive statistics and reliability estimates for the shared variables (in-class AI use and user attitudes) are identical to those reported in Fan et al. [61]; they are reproduced in Table 1-5 for the reader’s convenience, with appropriate citation. **(Section 4.1)**

Taking Table 1 as an example, the changes before and after are shown below (similar citation annotations have been applied across Tables 1-5):

**Original Table 1: Cronbach’s α coefficients of the scales**

| **Variable** | **CU** | **CE** | **BE** | **EE** | **AT** |
| --- | --- | --- | --- | --- | --- |
| Number of Items | 5 | 4 | 3 | 4 | 3 |
| Cronbach's α | .843 | .891 | .821 | .845 | .884 |

**Revised Table 1: Cronbach’s α coefficients of the scales**

| **Variable** | **CU** | **PCEB** | **PBEB** | **PEEB** | **AT** |
| --- | --- | --- | --- | --- | --- |
| Number of Items | 5 | 4 | 3 | 4 | 3 |
| Cronbach's α | .843 a | .891 | .821 | .845 | .884 a |

**Note: Symbol a refers to data obtained from Fan et al. [61].**

We hope that these revisions fully address your concerns and meet the high standards of PLOS ONE. Thank you once again for your constructive guidance.

**Sincerely,**

**The Authors**
