# Supplementary material for: In-class AI usage and university students’ attitudes: The mediating role of perceived learning engagement benefits
Source: PLoS One. 2026 Aug 4;21(8):e0355490. doi: 10.1371/journal.pone.0355490 (PMC13436728; doi:10.1371/journal.pone.0355490)
Supplement: Peer review history

# Peer review: Decision Letter 0

**DOI:** 10.1371/journal.pone.0355490.r001

**Authors:** Avanti Dey

15 Apr 2026

PONE-D-26-08042

The mediating role of learning engagement between university students' in-class AI usage behavior and attitudes: Are there differences across dimensions of engagement?

PLOS One

Dear Dr. Li,

Thank you for submitting your manuscript to PLOS ONE. After careful consideration, we feel that it has merit but does not fully meet PLOS ONE’s publication criteria as it currently stands. Therefore, we invite you to submit a revised version of the manuscript that addresses the points raised during the review process.

Please submit your revised manuscript by May 30 2026 11:59PM. If you will need more time than this to complete your revisions, please reply to this message or contact the journal office at plosone@plos.org. When you're ready to submit your revision, log on to https://www.editorialmanager.com/pone/ and select the 'Submissions Needing Revision' folder to locate your manuscript file.

Please include the following items when submitting your revised manuscript:

- A letter that responds to each point raised by the academic editor and reviewer(s). You should upload this letter as a separate file labeled 'Response to Reviewers'.
- A marked-up copy of your manuscript that highlights changes made to the original version. You should upload this as a separate file labeled 'Revised Manuscript with Track Changes'.
- An unmarked version of your revised paper without tracked changes. You should upload this as a separate file labeled 'Manuscript'.

If you would like to make changes to your financial disclosure, please include your updated statement in your cover letter. Guidelines for resubmitting your figure files are available below the reviewer comments at the end of this letter.

If applicable, we recommend that you deposit your laboratory protocols in protocols.io to enhance the reproducibility of your results. Protocols.io assigns your protocol its own identifier (DOI) so that it can be cited independently in the future. For instructions see: https://journals.plos.org/plosone/s/submission-guidelines#loc-laboratory-protocols. Additionally, PLOS ONE offers an option for publishing peer-reviewed Lab Protocol articles, which describe protocols hosted on protocols.io. Read more information on sharing protocols at https://plos.org/protocols?utm_medium=editorial-email&utm_source=authorletters&utm_campaign=protocols.

As the corresponding author, your ORCID iD is verified in the submission system and will appear in the published article. PLOS supports the use of ORCID, and we encourage all coauthors to register for an ORCID iD and use it as well. Please encourage your coauthors to verify their ORCID iD within the submission system before final acceptance, as unverified ORCID iDs will not appear in the published article. *Only*  the individual author can complete the verification step; PLOS staff *cannot*  verify ORCID iDs on behalf of authors.

We look forward to receiving your revised manuscript.

Kind regards,

Avanti Dey, PhD

Staff Editor

PLOS One

Journal Requirements:

When submitting your revision, we need you to address these additional requirements.

1. Please ensure that your manuscript meets PLOS ONE's style requirements, including those for file naming. The PLOS ONE style templates can be found at

https://journals.plos.org/plosone/s/file?id=wjVg/PLOSOne_formatting_sample_main_body.pdf and https://journals.plos.org/plosone/s/file?id=ba62/PLOSOne_formatting_sample_title_authors_affiliations.pdf

2. Please include your full ethics statement in the ‘Methods’ section of your manuscript file. In your statement, please include the full name of the IRB or ethics committee who approved or waived your study, as well as whether or not you obtained informed written or verbal consent. If consent was waived for your study, please include this information in your statement as well.

3. Thank you for stating the following financial disclosure:

This research was supported by the Fundamental Research Funds for the Central Universities, Key Teaching Reform Project of Beihang University, and Research and Development Fund Project for Postgraduate Education of Beihang University (JG2024030).

Please state what role the funders took in the study. If the funders had no role, please state: "The funders had no role in study design, data collection and analysis, decision to publish, or preparation of the manuscript."

If this statement is not correct you must amend it as needed.

Please include this amended Role of Funder statement in your cover letter; we will change the online submission form on your behalf.

4. Please upload a copy of Figure 1, 2, to which you refer in your text on page 10, 15. If the figure is no longer to be included as part of the submission please remove all reference to it within the text.

5. Please include a separate caption for each figure in your manuscript.

6. If the reviewer comments include a recommendation to cite specific previously published works, please review and evaluate these publications to determine whether they are relevant and should be cited. There is no requirement to cite these works unless the editor has indicated otherwise.

[Note: HTML markup is below. Please do not edit.]

Reviewers' comments:

Reviewer's Responses to Questions

**Comments to the Author**

1. Is the manuscript technically sound, and do the data support the conclusions?

The manuscript must describe a technically sound piece of scientific research with data that supports the conclusions. Experiments must have been conducted rigorously, with appropriate controls, replication, and sample sizes. The conclusions must be drawn appropriately based on the data presented.

Reviewer #1: Partly

**********

2. Has the statistical analysis been performed appropriately and rigorously?

Reviewer #1: Yes

**********

3. Have the authors made all data underlying the findings in their manuscript fully available?

The PLOS Data policy requires authors to make all data underlying the findings described in their manuscript fully available without restriction, with rare exception (please refer to the Data Availability Statement in the manuscript PDF file). The data should be provided as part of the manuscript or its supporting information, or deposited to a public repository. For example, in addition to summary statistics, the data points behind means, medians and variance measures should be available. If there are restrictions on publicly sharing data—e.g. participant privacy or use of data from a third party—those must be specified.

Reviewer #1: No

**********

4. Is the manuscript presented in an intelligible fashion and written in standard English?

PLOS ONE does not copyedit accepted manuscripts, so the language in submitted articles must be clear, correct, and unambiguous. Any typographical or grammatical errors should be corrected at revision, so please note any specific errors here.

Reviewer #1: Yes

**********

5. Review Comments to the Author

Please use the space provided to explain your answers to the questions above. You may also include additional comments for the author, including concerns about dual publication, research ethics, or publication ethics. (Please upload your review as an attachment if it exceeds 20,000 characters)

Reviewer #1: • The title is too long and overly descriptive, reducing clarity and impact. Phrase “Are there differences across dimensions of engagement?” appears exploratory rather than theory-driven.

• Convenience sampling from a single research-intensive university (H University) in China severely limits generalizability. The sample (N=287) is relatively small for the complexity of the structural equation model tested.

• The sample disproportionately represents science and engineering students (202 vs. 85 humanities/social sciences), yet no analysis examines whether findings differ by discipline. This is a significant omission given that AI usage patterns likely vary substantially across fields.

• No power analysis is reported to justify that N=287 is sufficient for the SEM with multiple mediators and Bayesian estimation.

• All variables (CU, CE, BE, EE, AT) were measured using self-report questionnaires from the same respondents at a single time point. This introduces substantial risk of common method variance, which the authors do not adequately address. The Harman's single-factor test or more rigorous approaches (e.g., marker variable technique) are absent.

• The study uses cross-sectional data but makes causal claims about mediation (e.g., "AI usage affected students' attitudes both directly and indirectly"). Without longitudinal or experimental design, the direction of causality is ambiguous. Reverse causation (positive attitudes → more AI usage) is equally plausible.

• The Attitudes scale (3 items) was "compiled independently by the researchers" with no reported validation beyond this study. The authors should provide evidence of prior validation or at minimum report item wording and pilot testing results.

• The In-Class AI Usage Behavior Scale asks about frequency ("Never" to "Very frequently") but does not distinguish types of AI use (e.g., generative AI for content creation vs. search-based AI for fact-checking). Different AI uses likely have different effects on engagement dimensions.

• Social desirability bias is unaddressed. Students may overreport "appropriate" AI usage or engagement levels.

• The paper invokes Self-Perception Theory (Bem, 1972), Cognitive Consistency Theory, and Affective Events Theory but does not integrate them into a coherent framework. How do these theories work together? Which predictions do they make about differential mediation across engagement dimensions?

• The authors claim Bayesian analysis is "more suitable for relatively small sample sizes" but provide no comparison with frequentist approaches or sensitivity analyses. The choice of priors is not specified, which is a fundamental omission in Bayesian work.

• The path from BE → AT (β = -0.074, CI [-0.205, 0.056]) is non-significant, yet the authors interpret this as "behavioral engagement operates through a mechanism distinct from that of cognitive and emotional engagement." This is over-interpretation of a null finding. The confidence interval includes zero; the correct conclusion is that no evidence was found for mediation, not that a distinct mechanism exists.

• No information is provided about missing data. Were there incomplete responses? How were they handled?

**********

6. PLOS authors have the option to publish the peer review history of their article (what does this mean?). If published, this will include your full peer review and any attached files.

If you choose “no”, your identity will remain anonymous but your review may still be made public.

**Do you want your identity to be public for this peer review?**  For information about this choice, including consent withdrawal, please see our Privacy Policy.

Reviewer #1: **Yes:** Fahd naveed Kausar

**********

[NOTE: If reviewer comments were submitted as an attachment file, they will be attached to this email and accessible via the submission site. Please log into your account, locate the manuscript record, and check for the action link "View Attachments". If this link does not appear, there are no attachment files.]

To ensure your figures meet our technical requirements, please review our figure guidelines: https://journals.plos.org/plosone/s/figures

You may also use PLOS’s free figure tool, NAAS, to help you prepare publication quality figures: https://journals.plos.org/plosone/s/figures#loc-tools-for-figure-preparation.

NAAS will assess whether your figures meet our technical requirements by comparing each figure against our figure specifications.

# Author comment: Author response to Decision Letter 1

**DOI:** 10.1371/journal.pone.0355490.r002

4 May 2026

Response to Reviewers

Dear Editors and Reviewers,

We would like to express our sincere gratitude to the Academic Editor and the Reviewer for their rigorous evaluation of our manuscript (PONE-D-26-08042) and their highly constructive feedback. We have carefully considered all the comments and have revised the manuscript accordingly. We believe that these revisions have substantially improved the theoretical foundation, methodological rigor, and clarity of our paper.

Below, we provide a point-by-point response to each comment. The original comments are presented in bold, followed by our responses and the corresponding modifications made in the manuscript.

Part I: Responses to Journal Requirements (Academic Editor)

1. Please ensure that your manuscript meets PLOS ONE's style requirements, including those for file naming.

●Response: Thank you for this reminder. We have carefully checked the revised submission package and ensured that the manuscript has been revised to comply with PLOS ONE’s style requirements, including the formatting of the manuscript and the naming of the uploaded files. We will submit the revised files using the journal’s required file labels and formats, including “Response to Reviewers,” “Revised Manuscript with Track Changes,” and “Manuscript.”

2. Please include your full ethics statement in the ‘Methods’ section of your manuscript file. In your statement, please include the full name of the IRB or ethics committee who approved or waived your study, as well as whether or not you obtained informed written or verbal consent. If consent was waived for your study, please include this information in your statement as well.

●Response: Thank you for the reminder. We have incorporated the full ethics statement into the 'Methods' section as requested. Specifically, the details regarding the ethical waiver from the Ethics Committee of Beihang University and the informed written consent procedures have been added to the end of Section 4.1 (Survey samples).

3. Thank you for stating the following financial disclosure:

This research was supported by the Fundamental Research Funds for the Central Universities, Key Teaching Reform Project of Beihang University, and Research and Development Fund Project for Postgraduate Education of Beihang University (JG2024030).

Please state what role the funders took in the study. If the funders had no role, please state: "The funders had no role in study design, data collection and analysis, decision to publish, or preparation of the manuscript."

If this statement is not correct you must amend it as needed.

Please include this amended Role of Funder statement in your cover letter; we will change the online submission form on your behalf.

●Response: Thank you for your guidance. The funders had no role in study design, data collection and analysis, decision to publish, or preparation of the manuscript. We have added this required statement to the "Funding" section at the end of the revised manuscript. Additionally, we have explicitly included this amended Role of Funder statement in the new cover letter accompanying this revised submission. Please kindly review it.

4. Please upload a copy of Figure 1, 2, to which you refer in your text on page 10, 15. If the figure is no longer to be included as part of the submission please remove all reference to it within the text.

●Response: Thank you for this reminder. We apologize for the confusion caused by the initial file naming. Following PLOS ONE’s guidelines, we have submitted the figures as separate files rather than embedding the images in the manuscript. The files previously labeled as "S1_Fig.tif" and "S2_Fig.tif" are indeed the "Figure 1" and "Figure 2" referred to in the text. To ensure clarity and consistency, we have now renamed these image files to Fig1.tif and Fig2.tif and re-uploaded them to the submission system as "Figure" items.

5. Please include a separate caption for each figure in your manuscript.

●Response: Thank you for this reminder. In accordance with the journal's formatting guidelines, a separate caption has been added for each figure in the revised manuscript. Specifically, we have moved the captions from the end of the manuscript directly into the main text, inserting them immediately after their first citation (in Section 3.4 and Section 5.4, respectively), and deleted the "(TIF)" text at the end of the captions. Please kindly review.

6. If the reviewer comments include a recommendation to cite specific previously published works, please review and evaluate these publications to determine whether they are relevant and should be cited. There is no requirement to cite these works unless the editor has indicated otherwise.

●Response: Thank you for your guidance. In our case, the reviewers did not recommend any specific publications for citation.

Part II: Responses to Reviewer #1

A. Responses to Specific Comments

1. The title is too long and overly descriptive, reducing clarity and impact. Phrase “Are there differences across dimensions of engagement?” appears exploratory rather than theory-driven.

●Response: Thank you very much for this helpful comment. We agree that the original title is somewhat lengthy and that the interrogative phrase at the end may make the study appear more exploratory than theoretically focused. To improve clarity and concision, we have revised the title. Revised title: “In-class AI usage and university students' attitudes: The mediating role of learning engagement”

2. Convenience sampling from a single research-intensive university (H University) in China severely limits generalizability. The sample (N=287) is relatively small for the complexity of the structural equation model tested.

●Response: We acknowledge the limitations regarding sampling and sample size. Using convenience sampling at a single institution indeed restricts the generalizability of our results. We have revised the "6.3 Research limitations and future directions" section to reflect this more accurately. Modification: In Section 6.3, we explicitly stated: "First, the participants in the research sample were limited to students from a single research-intensive university in China, and the sample size (N=287) was relatively small. This may limit the generalizability of the findings to students in other types of institutions or different cultural contexts."

3. The sample disproportionately represents science and engineering students (202 vs. 85 humanities/social sciences), yet no analysis examines whether findings differ by discipline. This is a significant omission given that AI usage patterns likely vary substantially across fields.

●Response: Thank you for your comment. Your insight is very reasonable—AI usage patterns may indeed differ among students from different academic disciplines. We conducted differential analyses on five core variables across disciplinary fields and found that three showed no significant differences, while two did show significant differences, while both with small effect sizes. This indicates that although the disciplinary differences are statistically significant, their practical impact is very small. Due to the unbalanced sample composition (overrepresentation of science and engineering students), we did not conduct direct group comparisons. (Added to Section 5.3)

4. No power analysis is reported to justify that N=287 is sufficient for the SEM with multiple mediators and Bayesian estimation.

●Response: Thank you for this important methodological point. We would like to clarify that traditional frequentist power analysis is not directly applicable to Bayesian estimation, because Bayesian inference does not rely on null hypothesis significance testing or the concept of statistical power in the Neyman–Pearson sense. Instead, the adequacy of sample size in Bayesian SEM is evaluated through MCMC convergence diagnostics and the precision of posterior estimates.

In our study, with N = 287 and 82 free parameters, the following evidence supports that the sample size is sufficient for stable estimation:

Convergence: All MCMC chains converged well, with PSR (Potential Scale Reduction Factor) ≤ 1.001, well below the recommended threshold of 1.05. Trace plots and autocorrelation plots showed no abnormal trends.

Effective Parameters: The pD (effective number of parameters) was 81.6, which is very close to the total number of free parameters (82), indicating that nearly all parameters were identified by the data rather than solely influenced by priors.

Estimation Precision: The 95% credible intervals for all parameters were reasonably narrow, providing sufficient precision for substantive interpretation.

Thus, while traditional power analysis was not performed, the reported Bayesian convergence diagnostics (added to Section 5.4) fully justify that N = 287 is adequate for the current model complexity.

5. All variables (CU, CE, BE, EE, AT) were measured using self-report questionnaires from the same respondents at a single time point. This introduces substantial risk of common method variance, which the authors do not adequately address. The Harman's single-factor test or more rigorous approaches (e.g., marker variable technique) are absent.

●Response: Thank you for this important methodological observation. In this study, Harman's single-factor test was used to examine common method bias. An unrotated principal component analysis on all measurement items revealed five factors with eigenvalues greater than 1.0, with the first factor accounting for 38.114% of the total variance, which is below the recommended threshold of 50% (Podsakoff et al., 2003). This indicates that common method bias is not a serious concern in this study and is unlikely to substantially distort the relationships among the variables. (Added to Section 4.3)

6. The study uses cross-sectional data but makes causal claims about mediation (e.g., "AI usage affected students' attitudes both directly and indirectly"). Without longitudinal or experimental design, the direction of causality is ambiguous. Reverse causation (positive attitudes → more AI usage) is equally plausible.

●Response: Thank you for this critical methodological observation. The reviewer is correct that cross-sectional data cannot support strong causal claims about mediation. The original wording has been revised throughout the manuscript to avoid causal language. The following actions have been taken:

1.Revision of causal language. All instances of causal claims (e.g., "affected," "influenced," "impacted") have been replaced with non-causal, associative language appropriate for cross-sectional data (e.g., "was associated with," "predicted", "was related to,"). The abstract, results, and discussion sections have all been revised accordingly.

2.Limitations statement. A clear limitation has been added to the Discussion section (Section 6.3) explicitly stating that the cross-sectional design precludes causal inference and that longitudinal or experimental designs are needed to establish temporal precedence and causality.

7. The Attitudes scale (3 items) was "compiled independently by the researchers" with no reported validation beyond this study. The authors should provide evidence of prior validation or at minimum report item wording and pilot testing results.

●Response: We appreciate the reviewer's vigilance. The three attitude items were reviewed by three subject matter experts. The complete wording of the three attitude items is provided in the Appendix (S2 File). In this study, the Cronbach's α for the Attitudes scale was 0.884. Exploratory factor analysis (EFA) extracted a single factor with an eigenvalue of 2.443, accounting for 81.431% of the total variance. The standardized factor loadings of the three items ranged from 0.890 to 0.920, with no evidence of double loading. Together, these indices consistently demonstrate that this newly developed Attitudes scale has good reliability and validity, indicating that the instrument is suitable for use in subsequent research (Added to Section 4.2). Nevertheless, we acknowledge the lack of prior validation as a limitation and have noted this in the Limitations section (Section 6.3) of the revised manuscript.

8. The In-Class AI Usage Behavior Scale asks about frequency ("Never" to "Very frequently") but does not distinguish types of AI use (e.g., generative AI for content creation vs. search-based AI for fact-checking). Different AI uses likely have different effects on engagement dimensions.

●Response: We sincerely thank the reviewer for pointing out this important nuance. We agree that treating AI usage primarily as a frequency variable without differentiating the specific types of AI tools (e.g., generative vs. search-based) is a limitation of our measurement instrument, as different AI functionalities may distinctively impact learning engagement. To address this, we have added this point to the research limitations (Section 6.3) as a critical direction for future studies.

9. Social desirability bias is unaddressed. Students may overreport "appropriate" AI usage or engagement levels.

●Response: Thank you for this helpful comment. We agree that social desirability bias is a potential concern in questionnaire-based research of this kind, and that students may have a tendency to overreport their AI usage and engagement when responding to related questions. In the present study, the survey was conducted anonymously, participation was voluntary, and no personally identifiable information was collected, which may help reduce respondents’ pressure to provide socially desirable answers. To address this concern more explicitly, we have added a brief statement in the limitations section (Section 6.3).

10. The paper invokes Self-Perception Theory (Bem, 1972), Cognitive Consistency Theory, and Affective Events Theory but does not integrate them into a coherent framework. How do these theories work together? Which predictions do they make about differential mediation across engagement dimensions?

●Response: Thank you for this insightful comment. We agree that the original manuscript did not sufficiently integrate the three theoretical perspectives. We have now added an integrated framework ("three-layer progressive consistency framework") of the three perspectives in the research model section (Section 3.4) to clarify how these theories work together and predict the mediating effects of different engagement dimensions based on these theories.

11. The authors claim Bayesian analysis is "more suitable for relatively small sample sizes" but provide no comparison with frequentist approaches or sensitivity analyses. The choice of priors is not specified, which is a fundamental omission in Bayesian work.

●Response: We thank the reviewer for these important points. We apologize for this omission. In the revised manuscript (Section 4.3), we have added a detailed Prior Specification subsection. Bayesian estimation employed Mplus default diffuse priors: factor loadings, path coefficients, and intercepts followed a normal distribution with a mean of 0 and a variance of 10¹⁰, while residual variances and covariances were assigned non-informative inverse-gamma priors. To examine the sensitivity of posterior inference to prior specifications, we re-estimated the model by replacing the priors for all structural path coefficients with weakly informative normal priors N(0,1), keeping all other specifications unchanged. The results showed that the Bayesian inference in this study is robust and not sensitive to the choice of priors. Following the reviewer's suggestion, we also estimated the same model using maximum likelihood (ML) estimation as a comparative approach (added to Section 5.4). Critically, the direction and significance of the core paths were entirely consistent with the Bayesian results. This demonstrates that the conclusions of this study are robust and consistent across two different estimation approaches (Bayesian and frequentist ML), providing strong evidence for the cross-methodological robustness of our findings.

12. The path from BE → AT (β = -0.074, CI [-0.205, 0.056]) is non-significant, yet the authors interpret this as "behavioral engagement operates through a mech

Attachment Submitted filename: Response to Reviewers.docx

# Peer review: Decision Letter 1

**DOI:** 10.1371/journal.pone.0355490.r003

**Authors:** Jihua Dong

5 Jun 2026

PONE-D-26-08042R1

In-class AI usage and university students' attitudes: The mediating role of learning engagement

PLOS One

Dear Dr. Li,

Thank you for submitting your manuscript to PLOS ONE. After careful consideration, we feel that it has merit but does not fully meet PLOS ONE’s publication criteria as it currently stands. Therefore, we invite you to submit a revised version of the manuscript that addresses the points raised during the review process.

Please submit your revised manuscript by Jul 20 2026 11:59PM. If you will need more time than this to complete your revisions, please reply to this message or contact the journal office at plosone@plos.org. When you're ready to submit your revision, log on to https://www.editorialmanager.com/pone/ and select the 'Submissions Needing Revision' folder to locate your manuscript file.

Please include the following items when submitting your revised manuscript:

- A letter that responds to each point raised by the academic editor and reviewer(s). You should upload this letter as a separate file labeled 'Response to Reviewers'.
- A marked-up copy of your manuscript that highlights changes made to the original version. You should upload this as a separate file labeled 'Revised Manuscript with Track Changes'.
- An unmarked version of your revised paper without tracked changes. You should upload this as a separate file labeled 'Manuscript'.

If you would like to make changes to your financial disclosure, please include your updated statement in your cover letter. Guidelines for resubmitting your figure files are available below the reviewer comments at the end of this letter.

If applicable, we recommend that you deposit your laboratory protocols in protocols.io to enhance the reproducibility of your results. Protocols.io assigns your protocol its own identifier (DOI) so that it can be cited independently in the future. For instructions see: https://journals.plos.org/plosone/s/submission-guidelines#loc-laboratory-protocols. Additionally, PLOS ONE offers an option for publishing peer-reviewed Lab Protocol articles, which describe protocols hosted on protocols.io. Read more information on sharing protocols at https://plos.org/protocols?utm_medium=editorial-email&utm_source=authorletters&utm_campaign=protocols.

As the corresponding author, your ORCID iD is verified in the submission system and will appear in the published article. PLOS supports the use of ORCID, and we encourage all coauthors to register for an ORCID iD and use it as well. Please encourage your coauthors to verify their ORCID iD within the submission system before final acceptance, as unverified ORCID iDs will not appear in the published article. *Only*  the individual author can complete the verification step; PLOS staff *cannot*  verify ORCID iDs on behalf of authors.

We look forward to receiving your revised manuscript.

Kind regards,

Jihua Dong, Ph.D.

Academic Editor

PLOS One

**Journal Requirements:**

1. If the reviewer comments include a recommendation to cite specific previously published works, please review and evaluate these publications to determine whether they are relevant and should be cited. There is no requirement to cite these works unless the editor has indicated otherwise.

2. Please review your reference list to ensure that it is complete and correct. If you have cited papers that have been retracted, please include the rationale for doing so in the manuscript text, or remove these references and replace them with relevant current references. Any changes to the reference list should be mentioned in the rebuttal letter that accompanies your revised manuscript. If you need to cite a retracted article, indicate the article’s retracted status in the References list and also include a citation and full reference for the retraction notice.

[Note: HTML markup is below. Please do not edit.]

Reviewers' comments:

Reviewer's Responses to Questions

**Comments to the Author**

1. If the authors have adequately addressed your comments raised in a previous round of review and you feel that this manuscript is now acceptable for publication, you may indicate that here to bypass the “Comments to the Author” section, enter your conflict of interest statement in the “Confidential to Editor” section, and submit your "Accept" recommendation.

Reviewer #1: All comments have been addressed

Reviewer #2: (No Response)

Reviewer #3: (No Response)

**********

2. Is the manuscript technically sound, and do the data support the conclusions?

The manuscript must describe a technically sound piece of scientific research with data that supports the conclusions. Experiments must have been conducted rigorously, with appropriate controls, replication, and sample sizes. The conclusions must be drawn appropriately based on the data presented.

Reviewer #1: Yes

Reviewer #2: (No Response)

Reviewer #3: Partly

**********

3. Has the statistical analysis been performed appropriately and rigorously?

Reviewer #1: Yes

Reviewer #2: (No Response)

Reviewer #3: Yes

**********

4. Have the authors made all data underlying the findings in their manuscript fully available?

The PLOS Data policy requires authors to make all data underlying the findings described in their manuscript fully available without restriction, with rare exception (please refer to the Data Availability Statement in the manuscript PDF file). The data should be provided as part of the manuscript or its supporting information, or deposited to a public repository. For example, in addition to summary statistics, the data points behind means, medians and variance measures should be available. If there are restrictions on publicly sharing data—e.g. participant privacy or use of data from a third party—those must be specified.

Reviewer #1: Yes

Reviewer #2: (No Response)

Reviewer #3: Yes

**********

5. Is the manuscript presented in an intelligible fashion and written in standard English?

PLOS ONE does not copyedit accepted manuscripts, so the language in submitted articles must be clear, correct, and unambiguous. Any typographical or grammatical errors should be corrected at revision, so please note any specific errors here.

Reviewer #1: Yes

Reviewer #2: (No Response)

Reviewer #3: Yes

**********

6. Review Comments to the Author

Please use the space provided to explain your answers to the questions above. You may also include additional comments for the author, including concerns about dual publication, research ethics, or publication ethics. (Please upload your review as an attachment if it exceeds 20,000 characters)

Reviewer #1: The authors have fully met the requirements of the reviewer comments. The manuscript is ready for acceptance from a review-response perspective.

Reviewer #2: (No Response)

Reviewer #3: The mediators measure perceived benefit not engagement.

This is my key point. The instruments do not measure engagement. They measure agreement that AI enhances engagement. The authors say CE is "perception of whether in-class AI usage enhances cognitive engagement." Same for BE and EE.

So these are evaluative. AI-referential. They are close to perceived usefulness. The authors invoke TAM and perceived usefulness themselves on the mediator→attitude paths.

All same-method and highly overlapping constructs "I think AI boosts my thinking" predicting "I like AI" ishould be described more carefully.

Suggestion: relabel the constructs accurately throughout. They are perceived engagement benefits. Not engagement. and discuss how much indirect effect might be construct overlap rather than mechanism.

The revision adds a "three-layer progressive consistency framework." suggesting that it is developmental when in fact t is parallel. It is cross-sectional. Three mediators side by side. It cannot test temporal order.

So the framework's core claims are should be more tentatively claimed. Either scale the theory back to what the model evaluates. Or state clearly that the framework is an interpretive heuristic. Not a tested structure.

The "proportion mediated" figures (53.6% / 54.9%) are unstable with small effects. De-emphasize them.

One recommendation contradicts a finding. The paper says use AI to "stimulate behavioral engagement." But BE does not relate to attitudes. Reconcile this.

Control variables (gender, grade, major) are not fully specified in the structural model. This matters. The disciplinary differences for CE and BE were significant.

**********

7. PLOS authors have the option to publish the peer review history of their article (what does this mean?). If published, this will include your full peer review and any attached files.

If you choose “no”, your identity will remain anonymous but your review may still be made public.

**Do you want your identity to be public for this peer review?**  For information about this choice, including consent withdrawal, please see our Privacy Policy.

Reviewer #1: **Yes:** Dr Fahd Naveed Kasuar,

Affiliation: School of Education, Minhaj University Lahore, Pakistan

Designation: Chairman School of Education

Email: fahdnaveed1@hotmail.com

Reviewer #2: No

Reviewer #3: **Yes:** Guy Trainin

**********

[NOTE: If reviewer comments were submitted as an attachment file, they will be attached to this email and accessible via the submission site. Please log into your account, locate the manuscript record, and check for the action link "View Attachments". If this link does not appear, there are no attachment files.]

To ensure your figures meet our technical requirements, please review our figure guidelines: https://journals.plos.org/plosone/s/figures

You may also use PLOS’s free figure tool, NAAS, to help you prepare publication quality figures: https://journals.plos.org/plosone/s/figures#loc-tools-for-figure-preparation.

NAAS will assess whether your figures meet our technical requirements by comparing each figure against our figure specifications.

# Author comment: Author response to Decision Letter 2

**DOI:** 10.1371/journal.pone.0355490.r004

5 Jul 2026

Response to Reviewers

Dear Editors and Reviewers,

We would like to express our sincere gratitude to you for your rigorous evaluation of our manuscript (PONE-D-26-08042) and your highly constructive feedback. We have carefully considered all the comments and have revised the manuscript accordingly. We believe that these revisions have substantially improved the theoretical foundation, methodological rigor, and clarity of our paper.

Additionally, before proceeding to the point-by-point responses, we would like to briefly note that a “Declaration on Data Reuse" has been included at the very end of this document to clarify the relationship between this study and a recently published related work.

Below, we provide the point-by-point response to each comment. The original comments are followed by our responses and the corresponding modifications, which are highlighted in the revised manuscript.

Reviewer #1:

Comment 1. The authors have fully met the requirements of the reviewer comments. The manuscript is ready for acceptance from a review-response perspective.

●Response:

We sincerely thank the reviewer for the positive and encouraging assessment of our revisions. We are very glad to hear that our point-by-point responses have fully addressed all the concerns raised. We truly appreciate the reviewer’s time and constructive suggestions throughout this process, which have significantly improved the quality of our manuscript.

Reviewer #3:

Comment 1. The mediators measure perceived benefit not engagement. This is my key point. The instruments do not measure engagement. They measure agreement that AI enhances engagement. The authors say CE is "perception of whether in-class AI usage enhances cognitive engagement." Same for BE and EE. So these are evaluative. AI-referential. They are close to perceived usefulness. The authors invoke TAM and perceived usefulness themselves on the mediator→attitude paths. All same-method and highly overlapping constructs "I think AI boosts my thinking" predicting "I like AI" ishould be described more carefully.

Suggestion: relabel the constructs accurately throughout. They are perceived engagement benefits. Not engagement. and discuss how much indirect effect might be construct overlap rather than mechanism.

●Response:

We sincerely thank the reviewer for this crucial insight. We completely agree that our instruments measure the students' evaluative agreement that AI enhances their engagement, rather than objective engagement metrics. To accurately reflect this, we have systematically relabeled the constructs throughout the entire manuscript—including the title, abstract, hypotheses, instruments, and discussion—to "perceived cognitive engagement benefits (PCEB)", "perceived behavioral engagement benefits (PBEB)", and "perceived emotional engagement benefits (PEEB)".

Furthermore, we have followed your suggestion to explicitly discuss the issue of construct overlap. We added a new paragraph to the limitations section to address how the indirect effects might be partially attributable to this overlap or a generalized positive appraisal rather than a purely sequential mechanism.

●Modification:

(1) To ensure conceptual consistency from the very beginning, we have modified the title of the manuscript to replace the general term of learning engagement with our specific measured variable.

Modified title: In-class AI usage and university students' attitudes: The mediating role of perceived learning engagement benefits

(2) In Section 6.3 (Research limitations and future directions), we have added a new fifth paragraph between the fourth limitation and the subsequent discussion to explicitly acknowledge the measurement boundaries and address the potential issue of construct overlap.

Section 6.3 (Research limitations and future directions):

Fifth, the mediating constructs in this study measure students' perceived benefits of AI use regarding their engagement (PCEB, PBEB, PEEB), rather than engagement metrics. Since both the mediators and the outcome variable (attitudes) are self-report measures that reference AI and involve evaluative judgments, the observed indirect effects may be partly driven by construct overlap. Although our discriminant validity analysis confirmed the statistical distinctness of these constructs, future research would benefit from incorporating objective measures of engagement. (Section 6.3)

Comment 2. The revision adds a "three-layer progressive consistency framework." suggesting that it is developmental when in fact t is parallel. It is cross-sectional. Three mediators side by side. It cannot test temporal order. So the framework's core claims are should be more tentatively claimed. Either scale the theory back to what the model evaluates. Or state clearly that the framework is an interpretive heuristic. Not a tested structure.

●Response:

We greatly appreciate your rigorous evaluation of our theoretical framework and empirical model. You are correct that because our survey data is cross-sectional and the three mediators are evaluated in a parallel configuration, we cannot empirically verify a temporal order. We agree that our previous claims regarding a "developmental" or "progressive" trajectory were overstated. To address this, we have tempered our theoretical claims in Section 3.4. We revised the language to present a "three-dimensional theoretical framework" with "complementary interpretive angles" rather than a sequential or developmental process. Most importantly, we have added a clear statement explicitly defining the framework as an interpretive heuristic rather than a tested structure.

●Modification:

Section 3.4 (Research model):

It is important to note that, while this framework proposes multiple layers of psychological processing, the cross-sectional survey design does not allow for empirical tests of temporal sequence or causal direction among the dimensions. Accordingly, this theoretical framework should therefore be interpreted as a conceptual heuristic to map concurrent psychological channels rather than a temporally verified path structure. (Section 3.4)

Comment 3. The "proportion mediated" figures (53.6% / 54.9%) are unstable with small effects. De-emphasize them.

●Response:

We sincerely thank the reviewer for this valuable methodological advice. We fully agree that the "proportion mediated" statistic tends to be unstable when the indirect effect is relatively small.

Accordingly, we have removed the unstable figures. We have completely deleted all reports of the "proportion mediated" values (53.6% and 54.9%) from the revised manuscript.

•Modification:

The following sentence was deleted from Section 5.4 (Hypothesis testing):

"Of the significant indirect effects, CE and EE showed comparable mediating effects, accounting for 53.6% (95% CI [32.8%, 75.6%]) and 54.9% (95% CI [30.3%, 81.8%]) of the total indirect effects, respectively." (Deleted)

Comment 4. One recommendation contradicts a finding. The paper says use AI to "stimulate behavioral engagement." But BE does not relate to attitudes. Reconcile this.

●Response:

We sincerely appreciate the reviewer’s astute observation regarding this logical contradiction. You are correct that advising educators to "stimulate behavioral engagement" directly conflicted with our empirical finding that perceived behavioral engagement benefits do not significantly relate to or mediate student attitudes.

To resolve this contradiction and ensure strict alignment between our empirical evidence and pedagogical recommendations, we have systematically removed and rephrased the active advocacy for stimulating behavioral engagement in both the Practical Implications and Conclusions sections.

•Modification:

(1) In Section 6.2 (Practical implications), the third paragraph has been revised as follows:

A critical examination should be conducted on behavioral engagement in AI-supported classroom settings. The current study find that perceived behavioral engagement benefits cannot mediate the relationship between AI usage and attitudes toward AI, implying that superficial behavioral performance is insufficient to realize the educational value of AI. In teaching practice, AI should neither be used merely for the sake of doing so, nor should its usage be oversimplified, including such superficial practices as content generation and automated question answering [16]. Instead, emphasis should be placed on promoting deep learning and fostering emotional experiences. (Section 6.2)

(2) In Section 7 (Conclusions), the relevant pedagogical recommendation paragraph has been amended as follows:

Educational practice should move beyond the debate over whether AI use should be permitted, and instead the focus should be shifted to designing human-AI collaborative learning experiences that use AI as a catalyst for fostering deep learning and student development. This study recommends that educators integrate AI tools into their teaching design, optimize their guidance on in-class AI usage, and look beyond superficial behavioral participation to focus primarily on facilitating deep cognitive integration and fostering positive emotional experiences for their students. (Section 7)

Comment 5. Control variables (gender, grade, major) are not fully specified in the structural model. This matters. The disciplinary differences for CE and BE were significant.

● Response:

We sincerely thank the reviewer for identifying this critical issue. In response, we have made the following revisions:

We re-ran the structural equation model analysis. In the final adopted model, gender, grade, and major were specified as covariates predicting all endogenous variables (i.e., the independent variable, mediator, and dependent variable) in the structural model.

We compared the hypothesis testing results between the two models and confirmed that all hypothesized paths remain significant with unchanged directions before and after covariate adjustment (see the comparison below). This demonstrates that our findings are robust and not contingent upon whether covariates are controlled. Importantly, after controlling for major as a covariate, all our hypothesized relationships continue to hold, indicating that the observed effects are generalizable across disciplines.

● Modification:

In Section 5.4 (Hypothesis testing), we have updated the model description to explicitly clarify the inclusion of covariates, and the relevant paragraph has been revised as follows:

In this study, the hypotheses were tested using SEM conducted in Mplus 8.3. Bayesian analysis was employed with the Markov Chain Monte Carlo (MCMC) method for parameter estimation. The model employed two parallel chains over 40,000 iterations, with the first 10,000 iterations used as burn-in. Posterior samples were saved at intervals of every 10 iterations. Convergence diagnostics indicated that the final value of the potential scale reduction factor (PSRF) was 1.009, and the PSR values for all parameters were close to 1.000. This is well below the strict threshold of 1.05, thus verifying the reliability of the subsequent parameter estimation results [68, 69]. In addition, examination of the trace plots and autocorrelation plots for the parameters’ posterior distributions revealed no abnormal trends or high autocorrelation, further verifying the good model convergence. All analyses controlled for gender, grade, and major as covariates affecting the independent, mediating, and dependent variables. (Section 5.4)

Furthermore, the data in the corresponding tables and the path diagram have been updated to reflect the newly estimated coefficients after covariate adjustment:

1. Comparison of Table 6 (Before and After Modification)

Original Table 6: Standardized path coefficients of the SEM

Paths Coefficient estimates S.E. Lower 95%CI Upper 95%CI

CU→AT 0.197 0.058 0.083 0.308

CU→CE 0.338 0.061 0.212 0.453

CU→BE 0.272 0.066 0.139 0.397

CU→EE 0.374 0.061 0.249 0.489

CE→AT 0.380 0.068 0.242 0.509

BE→AT -0.074 0.067 -0.205 0.056

EE→AT 0.351 0.080 0.191 0.508

Revised Table 6: Standardized path coefficients of the SEM

Paths Coefficient estimates S.E. Lower 95%CI Upper 95%CI

CU→AT 0.185 0.058 0.071 0.299

CU→PCEB 0.338 0.062 0.212 0.454

CU→PBEB 0.284 0.066 0.151 0.410

CU→PEEB 0.383 0.062 0.254 0.499

PCEB→AT 0.384 0.069 0.244 0.516

PBEB→AT -0.072 0.068 -0.206 0.060

PEEB→AT 0.346 0.080 0.187 0.502

2. Comparison of Table 7 (Before and After Modification)

Original Table 7: Results of multiple mediating effect test (standardized)

Standardized effects Effect size proportion SE Estimates 95% CI

Lower Limit Higher Limit

Standardized total effects

CU→AT 100% 0.058 0.347 0.241 0.469

Standardized direct effects

CU→AT 56.8% 0.058 0.197 0.083 0.308

Standardized indirect effects

CU→CE→AT 28.8% 0.028 0.100 0.053 0.164

CU→BE→AT -4.3% 0.016 -0.015 -0.052 0.012

CU→EE→AT 29.7% 0.032 0.103 0.050 0.174

Total indirect effects 54.5% 0.039 0.189 0.120 0.273

Revised Table 7: Results of multiple mediating effect test (standardized)

Standardized effects Effect size proportion SE Estimates 95% CI

Lower Limit Higher Limit

Standardized total effects

CU→AT 100% 0.057 0.335 0.231 0.454

Standardized direct effects

CU→AT 55.2% 0.058 0.185 0.071 0.299

Standardized indirect effects

CU→PCEB→AT 53.8% 0.029 0.100 0.053 0.164

CU→PBEB→AT - 0.017 -0.015 -0.052 0.013

CU→PEEB→AT 54.8% 0.032 0.102 0.050 0.173

Total indirect effects 56.1% 0.039 0.188 0.119 0.273

3. Comparison of Path Diagram (Before and After Modification)

Original Fig 2: Mediating path diagram. (Before controlling for covariates)

Revised Fig 2: Mediating path diagram. (After controlling for covariates)

Journal Requirements:

1. If the reviewer comments include a recommendation to cite specific previously published works, please review and evaluate these publications to determine whether they are relevant and should be cited. There is no requirement to cite these works unless the editor has indicated otherwise.

●Response:

Thank you for the reminder. We confirm that no citations were recommended by the reviewers.

2. Please review your reference list to ensure that it is complete and correct. If you have cited papers that have been retracted, please include the rationale for doing so in the manuscript text, or remove these references and replace them with relevant current references. Any changes to the reference list should be mentioned in the rebuttal letter that accompanies your revised manuscript. If you need to cite a retracted article, indicate the article’s retracted status in the References list and also include a citation and full reference for the retraction notice.

●Response:

We thank the editor for this important reminder. Our responses are as follows:

(1) Retracted publications: We have carefully verified every entry in our reference list and confirm that none of the cited papers have been retracted. Therefore, no rationale for citing retracted articles is needed.

(2) Changes to the reference list: One replacement has been made. The original reference [61] has been substituted with a new publication. Details are provided below:

Removed (original [61]): Liu E, Dewaele JM, Wang J. Developing a short language classroom engagement scale (LCES) and linking it with needs satisfaction and achievement. System. 2024;120(1):103189. doi: 10.1016/j.system.2023.103189.

Added (new [61]): Fan W, Cheng L, Wang Y, Zhao Q, Li Y. In-class AI use and attitudes among university students: The different mediating roles of cognitive relief and cognitive offloading. Behav Sci. 2026;16(6):1014. doi: 10.3390/bs16061014.

The corresponding in-text citation has been updated accordingly, and all changes have been clearly marked in the revised manuscript for the editor's and reviewers' convenience.

Declaration on Data Reuse

In addition to addressing the reviewers' comments, we would also like to make a declaration regarding data reuse:

Data for the present study and Fan et al. (2026) (Available from: https://www.mdpi.com/2076-328X/16/6/1014� were sourced from the same large-scale survey project. The two investigations overlap on two constructs: in-class AI usage and user attitudes. Whereas Fan et a

Attachment Submitted filename: Response_to_Reviewers_auresp_2.docx

# Editor report: Decision Letter 2

**DOI:** 10.1371/journal.pone.0355490.r005

**Authors:** Jihua Dong

22 Jul 2026

In-class AI usage and university students' attitudes: The mediating role of perceived learning engagement benefits

PONE-D-26-08042R2

Dear Dr. Yao,

We’re pleased to inform you that your manuscript has been judged scientifically suitable for publication and will be formally accepted for publication once it meets all outstanding technical requirements.

Within one week, you’ll receive an e-mail detailing the required amendments. When these have been addressed, you’ll receive a formal acceptance letter and your manuscript will be scheduled for publication.

An invoice will be generated when your article is formally accepted. Please note, if your institution has a publishing partnership with PLOS and your article meets the relevant criteria, all or part of your publication costs will be covered. Please make sure your user information is up-to-date by logging into Editorial Manager at Editorial Manager® and clicking the ‘Update My Information' link at the top of the page. For questions related to billing, please contact billing support.

If your institution or institutions have a press office, please notify them about your upcoming paper to help maximize its impact. If they’ll be preparing press materials, please inform our press team as soon as possible -- no later than 48 hours after receiving the formal acceptance. Your manuscript will remain under strict press embargo until 2 pm Eastern Time on the date of publication. For more information, please contact onepress@plos.org.

Kind regards,

Jihua Dong, Ph.D.

Academic Editor

PLOS One

# Editor report: Acceptance letter

**DOI:** 10.1371/journal.pone.0355490.r006

**Authors:** Jihua Dong

PONE-D-26-08042R2

PLOS One

Dear Dr. Li,

I'm pleased to inform you that your manuscript has been deemed suitable for publication in PLOS One. Congratulations! Your manuscript is now being handed over to our production team.

At this stage, our production department will prepare your paper for publication. This includes ensuring the following:

* All references, tables, and figures are properly cited

* All relevant supporting information is included in the manuscript submission,

* There are no issues that prevent the paper from being properly typeset

You will receive further instructions from the production team, including instructions on how to review your proof when it is ready. Please keep in mind that we are working through a large volume of accepted articles, so please give us a few days to review your paper and let you know the next and final steps.

Lastly, if your institution or institutions have a press office, please let them know about your upcoming paper now to help maximize its impact. If they'll be preparing press materials, please inform our press team within the next 48 hours. Your manuscript will remain under strict press embargo until 2 pm Eastern Time on the date of publication. For more information, please contact onepress@plos.org.

You will receive an invoice from PLOS for your publication fee after your manuscript has reached the completed accept phase. If you receive an email requesting payment before acceptance or for any other service, this may be a phishing scheme. Learn how to identify phishing emails and protect your accounts at https://explore.plos.org/phishing.

If we can help with anything else, please email us at customercare@plos.org.

Thank you for submitting your work to PLOS ONE and supporting open access.

Kind regards,

PLOS ONE Editorial Office Staff

on behalf of

Professor Jihua Dong

Academic Editor

PLOS One
